# Impact of different anesthetics on ischemia reperfusion injuries in patients undergoing hepatectomy: a network meta-analysis

**DOI:** 10.3389/fmed.2026.1607841

**Published:** 2026-02-05

**Authors:** Shaojuan Wang, Fangyuan Tian, Rui Zhang, Zehui Deng, Ling Zhou, Lin Ren, Fengbo Wu

**Affiliations:** Department of Pharmacy, West China Hospital, Sichuan University, Chengdu, China

**Keywords:** anesthetics, hepatectomy, liver function, network meta-analysis, propofol, sevoflurane

## Abstract

**Background:**

Hepatectomy, a commonly employed surgical approach for treating liver-related disorders, has emerged as the primary method for the radical treatment of early stage liver cancer. This network meta-analysis was conducted to evaluate the impacts of different anesthetics on ischemia reperfusion injuries in patients undergoing hepatectomy.

**Methods:**

Randomized controlled trials (RCTs) and cohort studies were identified through searches in four databases, PubMed, Embase, the Cochrane Library, and the Web of Science, up to February 12, 2025. Non-English studies were excluded, and this study was conducted in compliance with PRISMA-NMA guidelines. Bayesian network meta-analysis was employed based on the R 4.3.2 and Stata 15.1. The efficacy of each intervention was evaluated through SUCRA (surface under the cumulative ranking curve).

**Results:**

The analysis included 19 studies with a total of 1,319 participants. In terms of reducing aspartate transaminase (AST) levels, the combination of propofol and sevoflurane exhibited the highest effectiveness (SUCRAs: 71.82%), followed by the combination of propofol, isoflurane, and fentanyl (SUCRAs: 61.87%), and sevoflurane monotherapy (SUCRAs: 58.61%). The combination of propofol and sevoflurane (SUCRAs: 71.94%) ranked first for reducing ALT levels, followed by propofol monotherapy (SUCRAs: 61.71%) and the combination of propofol and sufentanil (SUCRAs: 58.59%).

**Conclusion:**

The combination of propofol and sevoflurane appear to be the most effective approach for reducing AST and ALT levels. This combination could serve as a favorable anesthetic strategy to limit hepatic enzyme elevations after hepatectomy, thus supporting liver function protection.

**Systematic review registration:**

https://www.crd.york.ac.uk/PROSPERO/home, identifier CRD42023472070.

## 1 Introduction

Hepatectomy, a commonly employed surgical approach for treating liver-related disorders, has emerged as the primary method for the radical treatment of early stage liver cancer (1, 2). With continuous advancements in safety measures for hepatectomy, an increasing number of patients may now benefit from this surgical option (3). Nonetheless, given the liver’s crucial function and extensive blood supply, the surgery inherently carries a significant risk of substantial bleeding. Bleeding not only poses a direct complication but also significantly increases the potential risks of developing other complications. During surgery, if bleeding occurs, it may result in ischemia of the liver and intestines, causing structural and functional damage to the liver cells. Such damage further impacts the liver’s metabolic and immune functions. Moreover, the extent of intraoperative bleeding has a critical impact (4) on the occurrence of postoperative complications and the prognosis of patients. Therefore, precise techniques for blood flow management are essential for ensuring the safety of patients undergoing hepatectomy.

Various techniques are commonly employed to reduce blood loss during hepatectomy, such as inflow occlusion by compressing the hepatoduodenal ligament or employing the Pringle maneuver. However, it is crucial to note that these approaches may lead to liver injury (5). Present strategies (6–8) aiming at mitigating liver injury involve preoperative ischemic preconditioning, intermittent Pringle maneuver, and pharmacological interventions. Pharmacological interventions provide liver protection through preoperative anesthetic preconditioning, further underscoring the importance of anesthetic selection for hepatectomy. Previous studies have indicated that commonly employed anesthetics, such as propofol and volatile anesthetics, have beneficial effects on liver function. For example, study conducted by Schmidt et al. revealed that isoflurane preconditioning increases the expression of hepatic heme oxygenase-1 (HO-1), thereby safeguarding the rat liver against ischemia reperfusion injury (9). Similarly, a retrospective study conducted by Mangus et al. concluded that both sevoflurane and desflurane exhibited protective effects on the liver (10). Furthermore, research carried out by Kim et al. exhibited that intravenous anesthesia combined with propofol significantly mitigated liver ischemia reperfusion injury in animal studies (11). Despite the observed hepatoprotective properties of these anesthetic agents, the conclusions regarding the properties of different agents remain inconsistent, and a unified consensus on the optimal hepatoprotective agent has yet to be reached. Ola et al. found that employing propofol or dexmedetomidine for anesthetic maintenance led to less postoperative liver cell damage in patients undergoing hepatectomy compared to desflurane (12). Another study by Feng et al. indicated that intravenous propofol anesthesia, as opposed to inhaled sevoflurane anesthesia, can moderately alleviate the body’s inflammatory response in patients with liver cancer. Furthermore, it demonstrated a milder inhibitory effect on T lymphocyte immune function and had a lesser impact on liver function compared to other agents (13). Ozgul et al. reported no significant difference in clinical outcomes between isoflurane and propofol (14). A randomized clinical trial conducted by Oladimeji et al. revealed that both isoflurane and propofol have minimal impact on liver enzymes in patients without liver disease, although significant increases in ALT and total bilirubin are observed in the propofol group (15). Conflicting conclusions regarding the impact of various agents on liver function have been observed across current studies, highlighting the limitations of existing systematic reviews and meta-analyses (8, 16). Although individual studies have demonstrated the effectiveness of specific interventions, an absence of direct comparisons has limited the establishment of a consensus. To address this issue, a network meta-analysis is essential for integrating information from both direct and indirect comparisons. This study aimed to compare the effects of different anesthetics on postoperative liver function in patients undergoing hepatectomy, offering a valuable reference for anesthetics selection in such population.

## 2 Methods

The study protocol was registered in the International Prospective Register of Systematic Reviews (PROSPERO) with an identification number of CRD42023455435, and followed the recommendations outlined in the Preferred Reporting Items for Systematic Reviews (PRISMA-NMA) guidelines, as well as the extension for NMA.

### 2.1 Search strategy

Randomized controlled trials (RCTs) and cohort studies published in English were identified through searches in four databases, including PubMed, Embase, the Cochrane Library, and Web of Science, from the inception of the databases up to February 12, 2025. The search terms were created by combining subject-specific terms with free-text terms. The medical subject headings employed included “hepatectomy,” “anesthesia,” “aspartate aminotransferase,” and “alanine transaminase.” The details of the search strategies are provided in Supplementary Appendix 1.

### 2.2 Inclusion and exclusion criteria

The inclusion criteria were as follows: (1) patients undergoing hepatectomy; (2) interventions consisting of either a single anesthetic agent or a combination of two or more anesthetic agents; (3) study types: randomized controlled trials or cohort studies; and (4) outcome measures: AST and/or ALT levels.

The following publications were excluded: (1) animal or cell experiments, case reports, scientific experimental designs, reviews, letters, editorials, conference papers; (2) studies with missing data or significant errors; (3) duplicates; or (4) studies lacking accessible full texts. Details of literature exclusions can be found in Supplementary Table 1.

### 2.3 Data extraction

The retrieved documents were imported into EndNote for management. Two researchers, Wang and Zhang, meticulously evaluated the papers based on their titles and abstracts in line with established criteria. Subsequently, the full texts were checked. In cases where disagreement arose between the two researchers, discussions were held to reach a consensus. If necessary, a third researcher, Deng, was brought in for consultation. To ensure accurate data extraction, two researchers independently used Excel tables to gather relevant information from the included studies. The data included the first author, publication year, country, duration of data collection, study type, presence of randomized and blinded design, intervention and control measures, sample size, patient demographics (age, disease), anesthesia method, and time points for outcome measurement.

### 2.4 Quality assessment

The Cochrane Risk of Bias Assessment Tool (RoB2.0) (17) was utilized to evaluate the quality of RCTs in terms of five critical domains: randomization bias, intervention bias, missing outcome data bias, outcome measurement bias, and selective reporting bias. The quality of each study was independently assessed by two researchers, who categorized each domain as “low risk,” “high risk,” or “some concerns.” Any disagreements arising during the review process were resolved through discussion or, if necessary, by a third researcher. The assessment results are visually represented in a risk of bias graph.

To evaluate the methodological quality of cohort studies, the Newcastle-Ottawa Scale (NOS) was employed. This scale encompasses cohort selection, comparability, and outcomes, with a score ranging from 0 to 9. Studies scoring 6 or above were deemed high-quality. Any disagreements were resolved through consultation with a senior researcher (18).

### 2.5 Certainty of evidence

Two researchers independently applied the GRADE method to assess the quality of evidence for each outcome, taking limitations, indirectness, inconsistency, imprecision, and publication bias into considerations. Discrepancies, if any, were addressed by a third author.

### 2.6 Statistical analysis

The weighted mean differences (MDs) along with 95% CIs were used to express AST and ALT levels. To address the heterogeneity among trials, a Bayesian hierarchical random effects model was initially utilized to compare various treatment options for patients undergoing hepatectomy (19, 20). The computations and graphical presentations were conducted with R 4.3.2 and Stata 15.1 software. Based on the random effects model, Markov chain Monte Carlo (MCMC) simulation was implemented through Bayesian inference in R 4.3.2 software, with iteration parameters set at 50,000 iterations and 20,000 annealing cycles, to explore the posterior distributions of the interrogated nodes (21–23). To assess local inconsistencies in outcomes with closed loops, the node splitting method was used. A network graph was employed to illustrate the relationships between different treatments. Additionally, a comparison-adjusted funnel plot was used to evaluate potential publication bias (24, 25). The surface under the cumulative ranking probability (SUCRA) values, ranging from 0 to 1, were used to rank the treatments, with higher SUCRA values indicating higher rankings of interventions (26, 27). To ensure result stability, two sensitivity analyses were conducted to exclude specific population characteristics: (1) studies involving cirrhosis were excluded to focus on the non-cirrhotic group, (2) studies lacking postoperative day one measurement data were removed, and (3) cohort studies were excluded. A league table was constructed to present the comparisons between each pair of interventions for every outcome.

## 3 Results

### 3.1 Literature search and screening process

**FIGURE 1:**
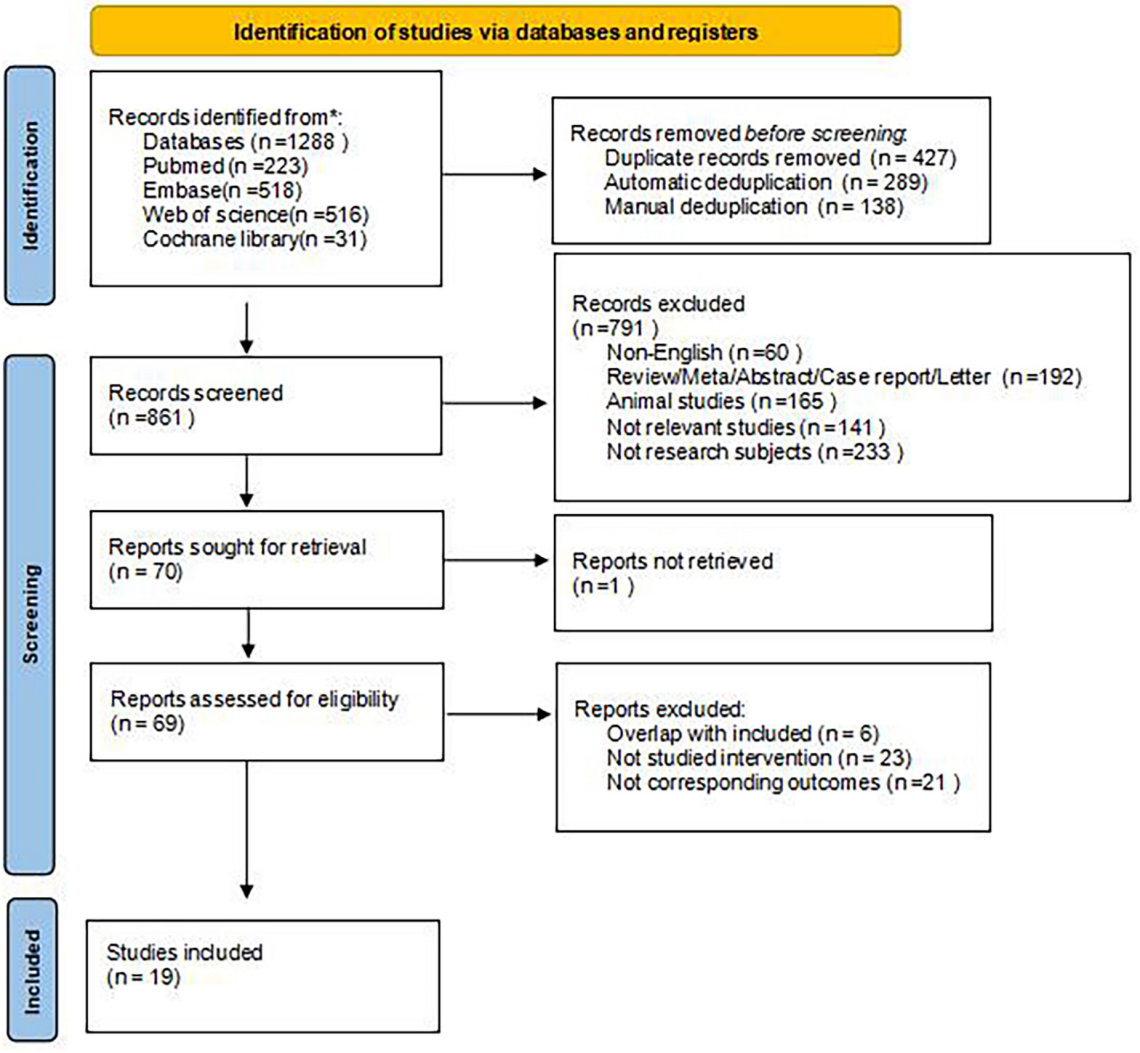
Literature screening flow chart.

Initially, 1,288 articles were identified, with 427 excluded during the initial screening. Subsequently, an additional 791 articles were excluded after reviewing their titles and abstracts. The full texts of the remaining articles were then checked based on established inclusion and exclusion criteria. In the end, 19 studies were selected for the analysis. The detailed screening process is illustrated in Figure 1.

### 3.2 Basic characteristics of the included studies

The analysis covers 19 studies (28–46) conducted across six countries: China, France, Switzerland, Saudi Arabia, Bulgaria, and Japan. These studies collectively involved 1,319 patients, comprising 797 males and 522 females with age ranging from 24.6 to 66.3 years. A total of seven different types of anesthetics and ten different treatment regimens were evaluated. The anesthetics used in these studies included isoflurane, sevoflurane, propofol, propofol + dexmedetomidine, propofol + remifentanil, propofol + sufentanil, propofol + isoflurane, propofol + sevoflurane, isoflurane + fentanyl, and propofol + isoflurane + fentanyl. The basic characteristics of the 19 included studies are shown in Supplementary Table 2.

### 3.3 Results of the methodological quality assessment of the included studies

**FIGURE 2:**
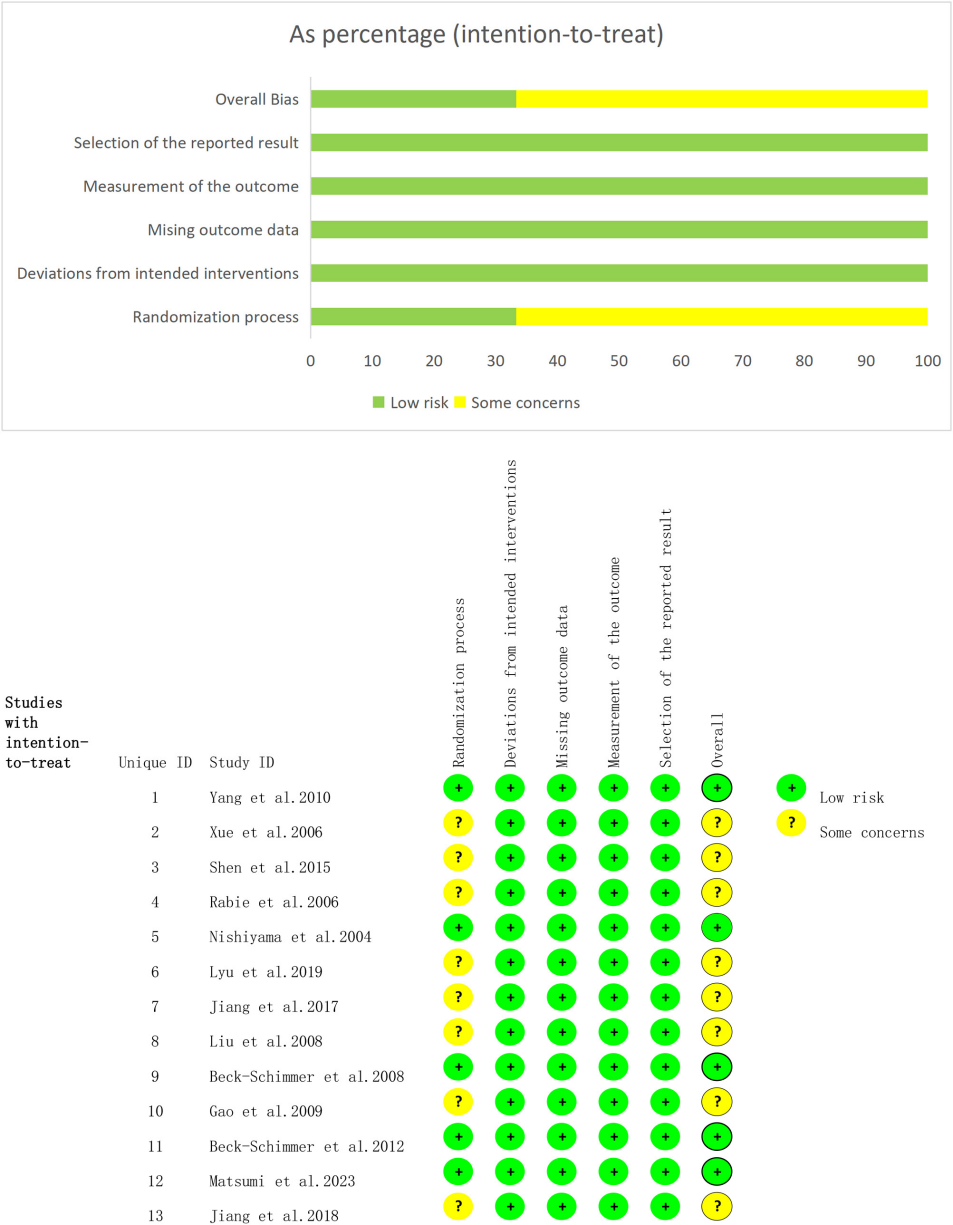
Risk bias assessment.

Figure 2 presents the results of bias risk assessment for the 13 RCTs. Regarding bias in the randomization, eight studies (29–31, 33–35, 37, 46) exhibited a potential risk due to insufficient concealment of group allocations, while the remaining five studies were classified as having a low risk. For bias in the deviation from established interventions, all studies were deemed to have a low risk. Moreover, all studies indicated a low risk of bias for missing outcome data and measurements. However, none of the studies exhibited a low risk of bias in selective reporting. Furthermore, all randomized trials had a low risk of bias in other sources. In conclusion, the overall risk of bias in RCTs was low.

The quality of the six cohort studies was evaluated based on three key criteria: cohort selection, comparability, and outcome measurement. All six studies received a score of 8, indicating that the overall quality of the cohort studies was high.

### 3.4 Network meta-analysis results

#### 3.4.1 Network graph

**FIGURE 3:**
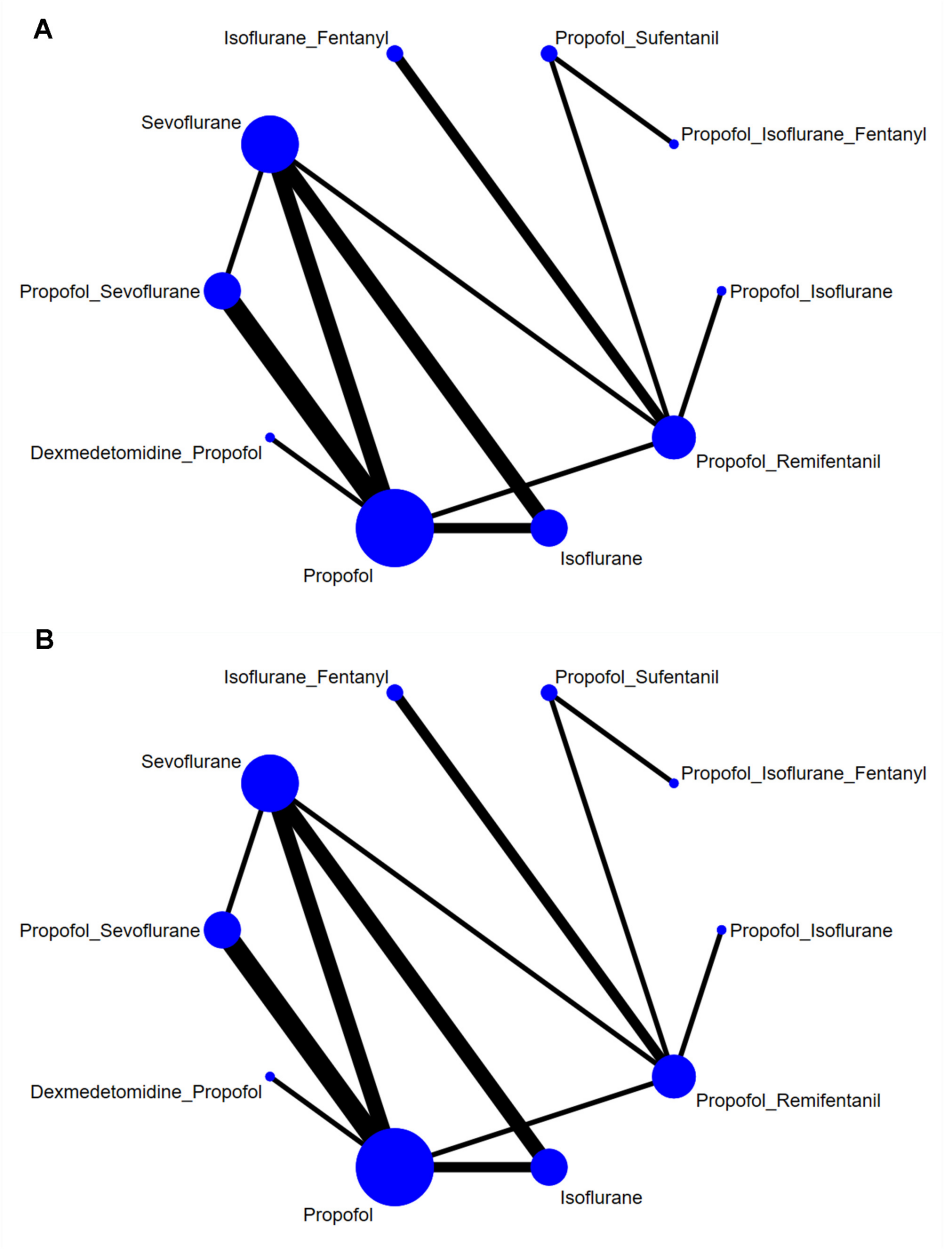
Network graph of **(A)** AST and **(B)** ALT.

This analysis included 19 studies involving 10 distinct interventions: isoflurane, sevoflurane, propofol, propofol + dexmedetomidine, propofol + remifentanil, propofol + sufentanil, propofol + isoflurane, propofol + sevoflurane, isoflurane + fentanyl, and propofol + isoflurane + fentanyl. Figure 3 presents the relationships between these interventions, with the width of the connecting lines reflecting the number of articles comparing each pair of interventions, and the size of each circle indicating the number of participants involved in each intervention.

Figure 3 demonstrates a closed loop. To investigate the effects within these closed loops, the node-splitting method was employed. The results indicated that all *P*-values exceed 0.05, suggesting an absence of local inconsistency, as shown in Supplementary Figure 1.

#### 3.4.2 AST

**FIGURE 4:**
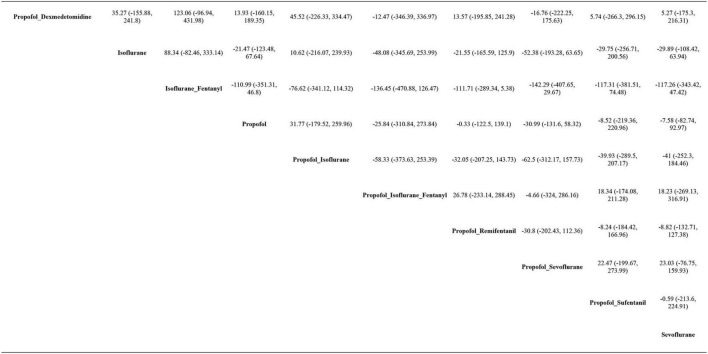
League table of AST.

AST levels were reported in all 19 studies, and pairwise comparisons of each intervention revealed no statistically significant differences in postoperative AST levels (as shown in Figure 4). According to the cumulative probability results, the three most effective interventions for reducing postoperative AST levels were propofol + sevoflurane (SUCRAs: 71.82%25), propofol + isoflurane + fentanyl (SUCRAs: 61.87%25), and sevoflurane (SUCRAs: 58.61%25), as indicated in Supplementary Figure 1 and Supplementary Table 3. The quality of evidence for these comparisons was considered moderate. Detailed data can be found in Supplementary Table 4.

#### 3.4.3 ALT

**FIGURE 5:**
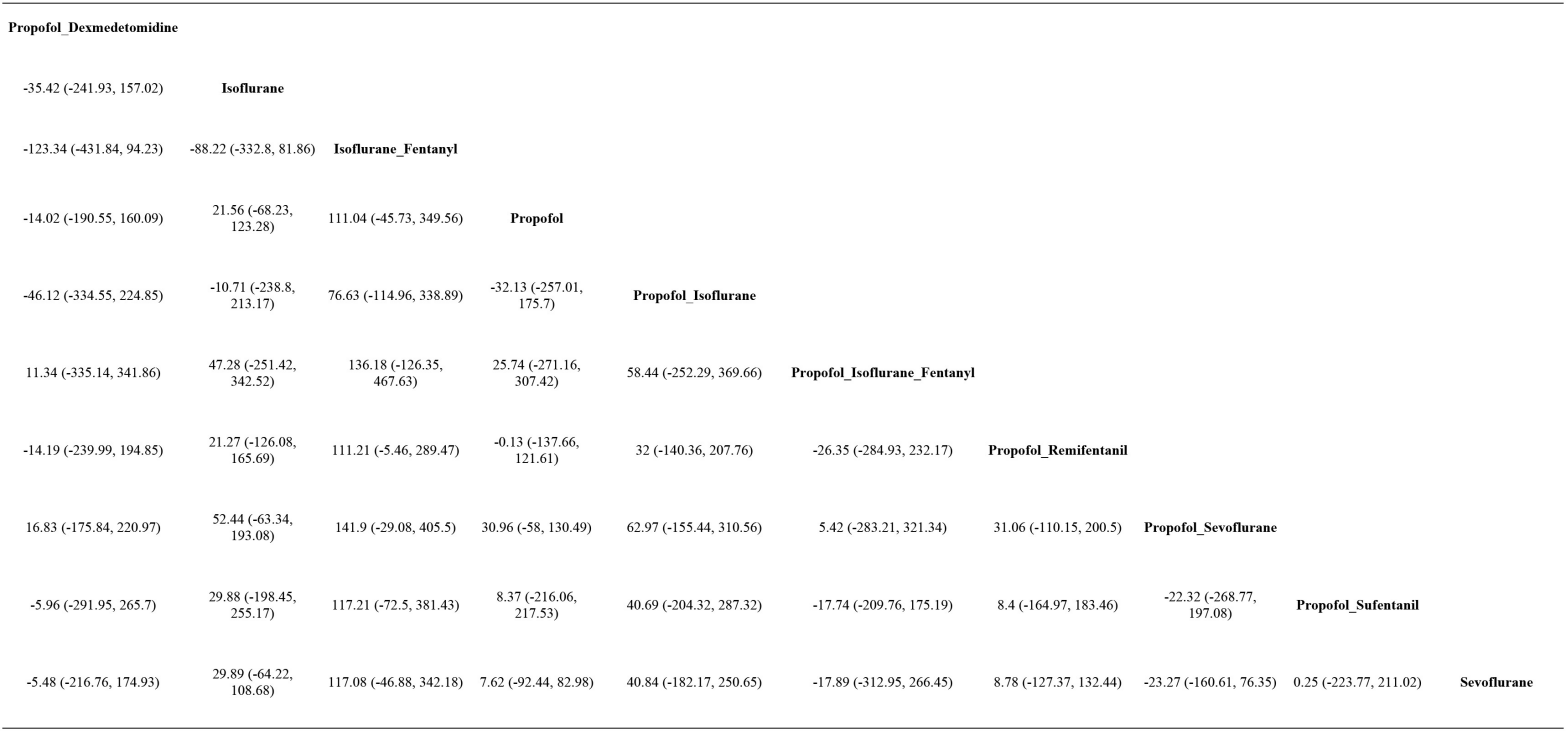
League table of ALT.

**FIGURE 6:**
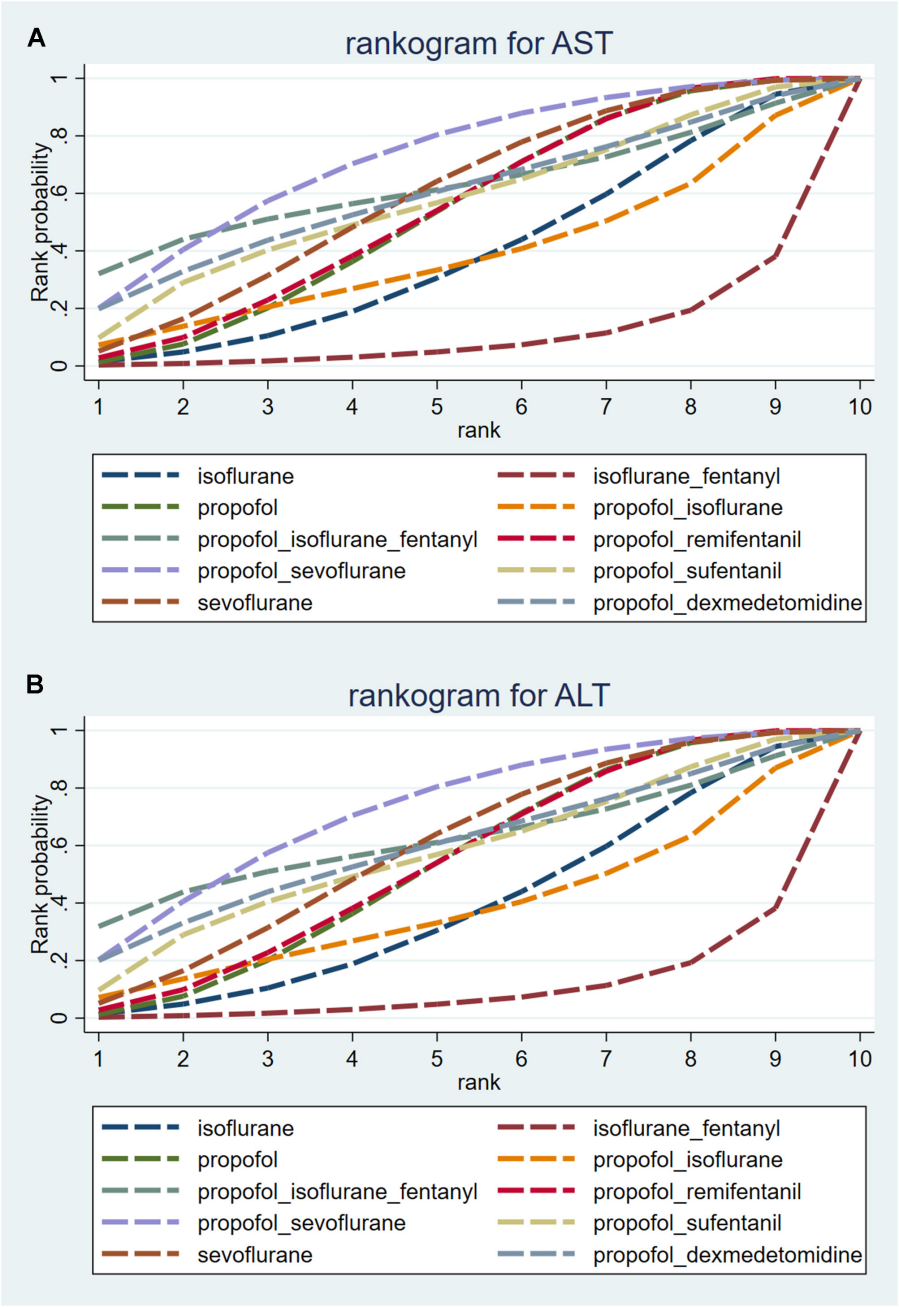
Probability line chart of **(A)** AST and **(B)** ALT.

ALT levels were reported in all 19 studies, and pairwise comparisons of each intervention revealed no statistically significant differences in postoperative ALT levels (as shown in Figure 5). According to the cumulative probability analysis, propofol + sevoflurane (SUCRAs: 71.94%), propofol (SUCRAs: 61.71%), and propofol + sufentanil (SUCRAs: 58.59%) are the three most effective interventions for reducing postoperative ALT levels (Figure 6 and Supplementary Table 3). The quality of evidence for these comparisons was considered moderate. Further data can be found in Supplementary Table 4.

### 3.5 Convergence analysis

Further convergence diagnostic analysis was conducted on the results. The potential scale reduction factor (PSRF) results indicated that the PSRFs of all effect indicators approached 1, demonstrating good model convergence (Supplementary Figures 2, 3).

### 3.6 Sensitivity analysis and regression analysis

The cirrhotic and non-cirrhotic populations were stratified, and due to sample size considerations, only the non-cirrhotic group was analyzed. The results showed minimal deviation from previous findings, demonstrating high stability (Supplementary Tables 5, 6). The sensitivity analysis on postoperative measurement timing was also conducted by exclusively extracting results from measurements on postoperative day one, which again confirmed robust stability (Supplementary Tables 7, 8). At the same time, we also excluded the cohort studies and conducted a sensitivity analysis only on RCTs. The results showed that the two interventions, propofol + isoflurane + fentanyl and propofol + sufentanil, could not form a loop. After removing these two interventions, we re-performed the sensitivity analysis. Changes in the included interventions led to altered SUCRA rankings, warranting cautious interpretation (Supplementary Tables 9, 10). To investigate whether the type of study and the method of anesthesia had an impact on the results, we added a regression analysis. The resulting confidence intervals all included 0, indicating that neither the type of study nor the method of anesthesia had a significant effect on the results (Supplementary Figures 4, 5).

### 3.7 Publication bias

**FIGURE 7:**
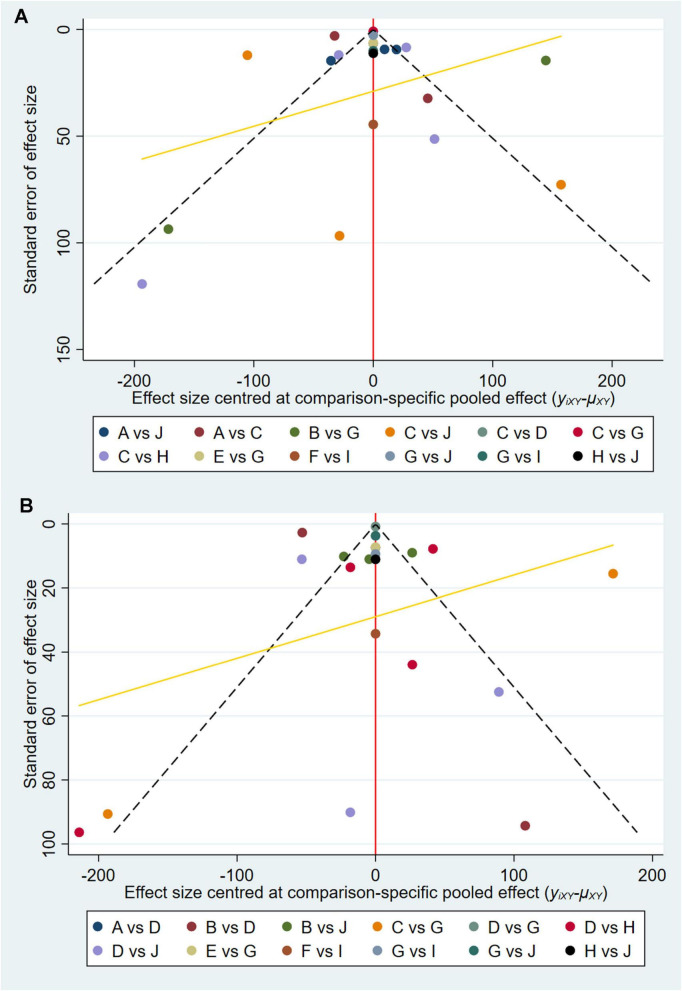
Funnel plot of **(A)** AST and **(B)** ALT.

Funnel plots were constructed to assess publication bias for all outcome indicators. The funnel plots for AST and ALT were asymmetrical, suggesting the potential presence of publication bias (Figure 7). However, further analysis based on Egger’s test revealed *P*-values of 0.854 for AST and 0.256 for ALT, both exceeding the threshold of 0.05, indicating the absence of publication bias.

## 4 Discussion

To the best of our knowledge, this study is the first network meta-analysis to evaluate the impact of different anesthetics on liver function in patients undergoing hepatectomy. Our analysis incorporates data from 13 eligible RCTs and 6 cohort studies. According to the cumulative probability ranking, propofol combined with sevoflurane may be regarded as the most effective approach for reducing both AST and ALT levels.

The results from the league table indicate that propofol combined with remifentanil may be more effective than isoflurane combined with fentanyl in reducing postoperative ALT levels. Propofol, known for its potent antioxidant properties, may effectively neutralize the detrimental effects of oxygen free radicals and protect liver cells from oxidative stress. A randomized study conducted by Martijn et al. has found that remifentanil combined with propofol, both of which are short-acting intravenous anesthetics with complementary pharmacokinetics for sedation and analgesia, is a promising option for intravenous anesthesia (47).

Hepatic ischemia-reperfusion injury is a complex pathophysiological event. Ischemic damage triggers inflammatory responses among hepatocytes, in addition to ATP depletion, mitochondrial dysfunction, and cellular oxidative stress, resulting in hepatocyte injury or death. During the subsequent reperfusion injury, liver metabolism is disturbed, triggering interconnected inflammatory cascades that further exacerbate hepatocellular damage. Different biomarkers can reflect the complex mechanisms of the injury. In this study, AST and ALT were selected as two of the biomarkers for liver injury.

The core mechanism of AST elevation is cellular damage or necrosis. When cells containing AST (particularly hepatocytes, cardiomyocytes, and myocytes) are subjected to injury, inflammation, or hypoxiaes) s cellular damage or necrosis. When cells containing AST (particularly hepan bias.attored in the cells will be released into circulation, thus elevating serum AST concentrations. Similar to AST but present at substantially higher concentrations in hepatocytes, ALTresent at slocalized in the cytoplasmhe cyresent at substantially higher concentrations in hepatocyte injury. When hepatocytes are injured, ALT within the cytoplasm is released into the bloodstream, leading to elevated serum ALT levels. In summary, elevated AST and ALT levels preliminarily indicate hepatocyte injury, with ALT demonstrating higher specificity than AST. According to the SUCRA ranking, the combination of propofol and sevoflurane is determined to be the most effective method for reducing AST and ALT levels. The league table results show no significant differences between these interventions, with overlapping intervals in the network meta-analysis league table, indicating that the efficacy of all interventions is comparable rather than any single intervention being superior. This underscores that clinical decision-making should integrate patient-specific characteristics, treatment safety, cost considerations, and physician expertise, rather than relying solely on the findings of this study.

Previous research by Olthof et al. and Schadde et al., has highlighted the widespread use of propofol due to its lower toxical, minimal liver metabolism, and absence of accumulation for hepatectomy (48, 49). Animal studies by Hao et al. have confirmed that propofol regulates MAPK6 expression via miR-133a-5p, providing protective effects against liver ischemia reperfusion injury (50). Sevoflurane, a novel inhalation anesthetic, does not irritate the respiratory tract and has no impact on hemodynamics, as it may block the n-methyl-D-aspartate receptors. Pan et al. have found that sevoflurane may be rapidly metabolized and excreted through the respiratory system once inhalation is stopped (51). Additionally, Xu et al. demonstrated that the combination use of propofol and sevoflurane has no significant effect on hemodynamics compared to using propofol alone, making this combination highly valuable for hepatectomy (45). The addition of sevoflurane has a minimal impact on hemodynamic stability and shows little effect on the inflammatory response. Furthermore, this combination does not worsen liver function, coagulation, or cognitive function, as reported by Xu et al. Moreover, the combination use of propofol and sevoflurane does not induce liver injury (52). A meta-analysis by Qiu et al. found that propofol, compared to inhalation anesthesia, may provide better postoperative analgesia within 24 h, with a lower incidence of nausea and vomiting (53). Considering both postoperative liver function and analgesic efficacy, the combination use of propofol and sevoflurane may represent an optimal choice.

The study conducted by Nguyen et al. (41) reported high mortality rates across all three groups. Most of their participants are old and suffer from malignant tumors, which may have influenced the observed mortality and incidence rates.

Multiple studies are incorporated in this network meta-analysis. Shen et al. and Nishiyama et al. performed randomized trials on sevoflurane and isoflurane, respectively. These studies address different diseases: cirrhosis complicated by liver cancer in the study of Shen et al., and cirrhosis with varying severity levels in the study by Nishiyama et al. Despite their studies focus on distinct diseases, both studies report similar conclusions. It is important to note that the size of the lesion may influence the extent of hepatectomy, which in turn could directly impact postoperative transaminase levels. Therefore, the extent of hepatectomy and the severity of the disease might potentially affect the clinical outcomes of patients.

A randomized trial by Beck-Schimmer et al. (36) examined the effects of propofol combined with sevoflurane for drug pretreatment. In their study, propofol concentrations range from 2 to 4 μg/mL. This combination strategy significantly improves clinical outcomes and reduces overall and major complications. Similarly, Xu et al. conducted a study with a fixed propofol concentration of 3 μg/ml, which shows that propofol combined with sevoflurane has a minimal impact on hemodynamic stability, inflammatory response, and liver function. Despite the slight difference in propofol concentrations between the two studies, they similarly report that the combination use of propofol and sevoflurane results in better patient prognoses. These consistent findings suggest that propofol concentrations have a limited influence on patient outcomes. However, it is also possible that the difference in propofol concentrations is too minor to affect the outcomes. Furthermore, Xue et al. explored a propofol concentration ranging from 2.4 to 3.6 mg/kg/h, indicating that propofol combined with remifentanil has little effect on liver function and patients are more conscious. Conversely, Jiang et al. used a propofol concentration ranging from 7 to 10 mg/kg/h, revealing that propofol combined with remifentanil effectively maintains intraoperative blood pressure and circulatory system stability in older patients, reduces the release of inflammatory factors, and minimizes liver ischemia reperfusion injury. Between the two experiments, there is a notable difference in propofol concentrations, indicating that propofol concentrations may have little effect on the outcomes observed.

Age has been recognized as a critical factor influencing the effects of ischemic preconditioning, as demonstrated in the study conducted by Beck-Schimmer et al. (36). The research indicates that the response of liver cells to ischemic injury may differ between younger and older individuals. Furthermore, the findings by Beck-Schimmer et al. (38) showed that age, the extent of liver steatosis, and preoperative chemotherapy have no noticeable impact on outcomes. However, in the study conducted by Liao et al. (44), the participants are not categorized according to their specific characteristics. Considering the possible differences in physiological functions across different age groups and other factors, it is reasonable to hypothesize that age can affect the clinical outcomes of patients.

There are several limitations in our study. Firstly, the majority of participants in the included studies are from China, with only a small proportion from other countries, which may limit the generalizability of our findings to a more diverse population. Secondly, the number of studies included in our analysis is relatively limited, especially regarding intervention studies, potentially affecting the strength and reliability of our conclusions. Lastly, although randomized controlled studies are included, some articles do not use proper blinding methods, which could introduce bias into our results. Additionally, there are no apparent differences in gender among the 18 articles included, which might have implications for the overall conclusions. This aspect should be considered in future studies to provide more insights. Additionally, certain limitations exist in the extracted data from the studies included in our analysis. Thus, it is impossible for us to conduct subgroup analyses, which may have an impact on our findings, particularly in terms of factors such as hepatectomy scope, liver diseases, anesthetic dosages, outcome measurement timing, and outcome selection. These factors may exert certain influence on the outcomes. While AST and ALT levels serve as markers of postoperative liver injury, clinical outcomestcomes. While AST and ALT levels should be considered in future studies 3 meta.ating that neither the type of study nor the ers set at 50,000 iterations and 20,000 annealas surrogate markers for the severity of ischemia-reperfusion injury (IRI) has certain limitations. However, due to the limited number of papers addressing these outcomes, further analysis was not possible. Therefore, future research is necessary to address these limitations and further validate our findings.

Our research indicates that propofol combined with remifentanil appears to be more effective than isoflurane combined with fentanyl in reducing ALT levels. Furthermore, the combination use of propofol and sevoflurane may be the most effective method for reducing both AST and ALT levels. Postoperative AST and ALT levels serve as partial indicators of liver function impairment. Considering previous findings, the combination use of propofol and sevoflurane is recommended for hepatectomy to minimize liver function damage.

However, due to limited number and quality of the studies included in our analysis, further high-quality, large-scale, double-blind, randomized controlled trials are necessary to validate our findings.

## Data Availability

The original contributions presented in this study are included in the article/Supplementary material, further inquiries can be directed to the corresponding author.
