# Supplementary material for: Impact of different anesthetics on ischemia reperfusion injuries in patients undergoing hepatectomy: a network meta-analysis
Source: Front Med (Lausanne). 2026 Feb 5;13:1607841. doi: 10.3389/fmed.2026.1607841 (PMC12916711; doi:10.3389/fmed.2026.1607841)
Supplement: Supplementary file 1 [file Data_Sheet_1.docx]

**Supplementary Materials**

**Appendix 1** Search strategies

**Supplementary Table S1** Details of reasons for article exclusion

**Supplementary Table S2** Baseline characteristics

**Supplementary Table S3** Scura ranking table

**Supplementary Table S4** Grade assessments

**Supplementary Table S5** Scura ranking table of non-cirrhotic

**Supplementary Table S6a** League Table for Sensitivity Analysis 1 (AST)

**Supplementary Table S6b** League Table for Sensitivity Analysis 1 (ALT)

**Supplementary Table S7** Scura ranking table of enzyme measurement time

**Supplementary Table S8a** League Table for Sensitivity Analysis 2 (AST)

**Supplementary Table S8b** League Table for Sensitivity Analysis 2 (ALT)

**Supplementary Table S9** Scura ranking table of RCT

**Supplementary Table S10a** League Table for Sensitivity Analysis 3 (AST)

**Supplementary Table S10b** League Table for Sensitivity Analysis 3 (ALT)

**Supplementary Figure S1** Results of (A) AST and (B) ALT node splitting analysis

**Supplementary Figure S2** Results of (A) AST and (B) ALT node splitting analysis

**Supplementary Figure S3** Results of Convergence diagnostics for MCMC (ALT)

**Supplementary Figure S4** Meta-regression analysis for type of study

**Supplementary Figure S5** Meta-regression analysis for anesthesia method

**Appendix 1** Search strategies

**PubMed**

| **Search number** | **Query** | **Results** |
| --- | --- | --- |
| 14 | (((("Hepatectomy"[Mesh]) OR ((Hepatectomy[Title/Abstract]) OR (Hepatectomies[Title/Abstract]))) OR ((liver[Title/Abstract] OR hepatic[Title/Abstract] OR hepato[Title/Abstract]) AND (resection[Title/Abstract] OR surgery[Title/Abstract]))) AND ((Anesthesia[Title/Abstract] OR Anesthetic*[Title/Abstract] OR anaesthesia[Title/Abstract] OR anaesthetic[Title/Abstract] OR anesthetization[Title/Abstract] OR autoanaesthesia[Title/Abstract] OR autoanesthesia[Title/Abstract] OR narcosis[Title/Abstract] OR neuroanaesthesia[Title/Abstract] OR Neuroanesthesia[Title/Abstract]) OR ("Anesthesia"[Mesh]))) AND (((("Aspartate Amainotransferases"[Mesh]) OR (Aspartate Aminotransferase[Title/Abstract] OR Aspart* Aminotransferase*[Title/Abstract] OR Aspart* Transaminase[Title/Abstract] OR Aspartate Apoaminotransferase[Title/Abstract] OR aspartate amino transferase[Title/Abstract] OR AST[Title/Abstract] OR GOT[Title/Abstract] OR SGOT[Title/Abstract] OR glutam* oxal* transaminase[Title/Abstract] OR glutamate oxaloacetate*[Title/Abstract] OR glutamate oxalacetate transaminase[Title/Abstract] OR glutam* oxaloacetic*[Title/Abstract] OR glutamatoxalacetate transaminase[Title/Abstract] OR glutamic aspartic*[Title/Abstract])) OR (Alanine Transaminase[Title/Abstract] OR SGPT[Title/Abstract] OR Alanin* aminotransferase[Title/Abstract] OR alanine amino transferase[Title/Abstract] OR alanine* transaminase[Title/Abstract] OR alanine transpeptidase[Title/Abstract] OR glutamate pyruvate*[Title/Abstract] OR glutamic pyruv* transaminase[Title/Abstract] OR glutamic pyruvic aminotransferase[Title/Abstract] OR glutamopyruvic transaminase[Title/Abstract] OR GPT[Title/Abstract])) OR ("Alanine Transaminase"[Mesh])) | 223 |
| 13 | ((("Aspartate Aminotransferases"[Mesh]) OR (Aspartate Aminotransferase[Title/Abstract] OR Aspart* Aminotransferase*[Title/Abstract] OR Aspart* Transaminase[Title/Abstract] OR Aspartate Apoaminotransferase[Title/Abstract] OR aspartate amino transferase[Title/Abstract] OR AST[Title/Abstract] OR GOT[Title/Abstract] OR SGOT[Title/Abstract] OR glutam* oxal* transaminase[Title/Abstract] OR glutamate oxaloacetate*[Title/Abstract] OR glutamate oxalacetate transaminase[Title/Abstract] OR glutam* oxaloacetic*[Title/Abstract] OR glutamatoxalacetate transaminase[Title/Abstract] OR glutamic aspartic*[Title/Abstract])) OR (Alanine Transaminase[Title/Abstract] OR SGPT[Title/Abstract] OR Alanin* aminotransferase[Title/Abstract] OR alanine amino transferase[Title/Abstract] OR alanine* transaminase[Title/Abstract] OR alanine transpeptidase[Title/Abstract] OR glutamate pyruvate*[Title/Abstract] OR glutamic pyruv* transaminase[Title/Abstract] OR glutamic pyruvic aminotransferase[Title/Abstract] OR glutamopyruvic transaminase[Title/Abstract] OR GPT[Title/Abstract])) OR ("Alanine Transaminase"[Mesh]) | 144,259 |
| 12 | "Alanine Transaminase"[Mesh] | 34,203 |
| 11 | Alanine Transaminase[Title/Abstract] OR SGPT[Title/Abstract] OR Alanin* aminotransferase[Title/Abstract] OR alanine amino transferase[Title/Abstract] OR alanine* transaminase[Title/Abstract] OR alanine transpeptidase[Title/Abstract] OR glutamate pyruvate*[Title/Abstract] OR glutamic pyruv* transaminase[Title/Abstract] OR glutamic pyruvic aminotransferase[Title/Abstract] OR glutamopyruvic transaminase[Title/Abstract] OR GPT[Title/Abstract] | 57,541 |
| 10 | Aspartate Aminotransferase[Title/Abstract] OR Aspart* Aminotransferase*[Title/Abstract] OR Aspart* Transaminase[Title/Abstract] OR Aspartate Apoaminotransferase[Title/Abstract] OR aspartate amino transferase[Title/Abstract] OR AST[Title/Abstract] OR GOT[Title/Abstract] OR SGOT[Title/Abstract] OR glutam* oxal* transaminase[Title/Abstract] OR glutamate oxaloacetate*[Title/Abstract] OR glutamate oxalacetate transaminase[Title/Abstract] OR glutam* oxaloacetic*[Title/Abstract] OR glutamatoxalacetate transaminase[Title/Abstract] OR glutamic aspartic*[Title/Abstract] | 104,212 |
| 9 | "Aspartate Aminotransferases"[Mesh] | 32,073 |
| 8 | ((("Hepatectomy"[Mesh]) OR ((Hepatectomy[Title/Abstract]) OR (Hepatectomies[Title/Abstract]))) OR ((liver[Title/Abstract] OR hepatic[Title/Abstract] OR hepato[Title/Abstract]) AND (resection[Title/Abstract] OR surgery[Title/Abstract]))) AND ((Anesthesia[Title/Abstract] OR Anesthetic*[Title/Abstract] OR anaesthesia[Title/Abstract] OR anaesthetic[Title/Abstract] OR anesthetization[Title/Abstract] OR autoanaesthesia[Title/Abstract] OR autoanesthesia[Title/Abstract] OR narcosis[Title/Abstract] OR neuroanaesthesia[Title/Abstract] OR Neuroanesthesia[Title/Abstract]) OR ("Anesthesia"[Mesh])) | 2,892 |
| 7 | (Anesthesia[Title/Abstract] OR Anesthetic*[Title/Abstract] OR anaesthesia[Title/Abstract] OR anaesthetic[Title/Abstract] OR anesthetization[Title/Abstract] OR autoanaesthesia[Title/Abstract] OR autoanesthesia[Title/Abstract] OR narcosis[Title/Abstract] OR neuroanaesthesia[Title/Abstract] OR Neuroanesthesia[Title/Abstract]) OR ("Anesthesia"[Mesh]) | 405,370 |
| 6 | "Anesthesia"[Mesh] | 212,019 |
| 5 | Anesthesia[Title/Abstract] OR Anesthetic*[Title/Abstract] OR anaesthesia[Title/Abstract] OR anaesthetic[Title/Abstract] OR anesthetization[Title/Abstract] OR autoanaesthesia[Title/Abstract] OR autoanesthesia[Title/Abstract] OR narcosis[Title/Abstract] OR neuroanaesthesia[Title/Abstract] OR Neuroanesthesia[Title/Abstract] | 334,606 |
| 4 | (("Hepatectomy"[Mesh]) OR ((Hepatectomy[Title/Abstract]) OR (Hepatectomies[Title/Abstract]))) OR ((liver[Title/Abstract] OR hepatic[Title/Abstract] OR hepato[Title/Abstract]) AND (resection[Title/Abstract] OR surgery[Title/Abstract])) | 111,864 |
| 3 | (liver[Title/Abstract] OR hepatic[Title/Abstract] OR hepato[Title/Abstract]) AND (resection[Title/Abstract] OR surgery[Title/Abstract]) | 88,691 |
| 2 | (Hepatectomy[Title/Abstract]) OR (Hepatectomies[Title/Abstract]) | 28,313 |
| 1 | "Hepatectomy"[Mesh] | 36,168 |

**Embase**

| No. | Query | Results | Date |
| --- | --- | --- | --- |
| #16 | #10 AND #15 | 518 | 12-Feb-25 |
| #15 | #11 OR #12 OR #13 OR #14 | 312308 | 12-Feb-25 |
| #14 | alanine AND transaminase OR sgpt OR (alanin* AND aminotransferase) OR (alanine AND amino AND transferase) OR (alanine* AND transaminase) OR (alanine AND transpeptidase) OR (glutamate AND pyruvate*) OR (glutamic AND pyruv* AND transaminase) OR (glutamic AND pyruvic AND aminotransferase) OR (glutamopyruvic AND transaminase) OR gpt | 206521 | 12-Feb-25 |
| #13 | 'alanine aminotransferase'/exp | 165466 | 12-Feb-25 |
| #12 | aspartate?aminotransferase OR aspart*?aminotransferase* OR aspart*?transaminase OR aspartate?apoaminotransferase OR aspartate?amino?transferase OR ast OR got OR sgot OR glutam*?oxal*?transaminase OR glutamate?oxaloacetate* OR glutamate?oxalacetate?transaminase OR glutam*?oxaloacetic* OR glutamatoxalacetate?transaminase OR glutamic?aspartic* | 249171 | 12-Feb-25 |
| #11 | 'aspartate aminotransferase'/exp | 147306 | 12-Feb-25 |
| #10 | #6 AND #9 | 5258 | 12-Feb-25 |
| #9 | #7 OR #8 | 586452 | 12-Feb-25 |
| #8 | anesthesia:ab,ti OR anesthetic*:ab,ti OR anaesthesia:ab,ti OR anaesthetic:ab,ti OR anesthetization:ab,ti OR autoanaesthesia:ab,ti OR autoanesthesia:ab,ti OR narcosis:ab,ti OR neuroanaesthesia:ab,ti OR neuroanesthesia:ab,ti | 420002 | 12-Feb-25 |
| #7 | 'anesthesia'/exp | 432028 | 12-Feb-25 |
| #6 | #1 OR #2 OR #5 | 166885 | 12-Feb-25 |
| #5 | #3 AND #4 | 131527 | 12-Feb-25 |
| #4 | resection:ab,ti OR surgery:ab,ti | 2296743 | 12-Feb-25 |
| #3 | liver:ab,ti OR hepatic:ab,ti OR hepato:ab,ti | 1478655 | 12-Feb-25 |
| #2 | hepatectomy:ab,ti OR hepatectomies:ab,ti | 36817 | 12-Feb-25 |
| #1 | 'hepatectomy'/exp | 71613 | 12-Feb-25 |

**Cochrane Library**

| ID | Search | Hits |
| --- | --- | --- |
| #1 | MeSH descriptor: [Hepatectomy] explode all trees | 1287 |
| #2 | (Hepatectomies or Hepatectomy):ti,ab,kw | 2206 |
| #3 | (liver OR hepatic OR hepato):ti,ab,kw | 70856 |
| #4 | (resection or surgery):ti,ab,kw | 277362 |
| #5 | #3 and #4 | 12699 |
| #6 | #1 or #2 or# 5 | 1619 |
| #7 | MeSH descriptor: [Anesthesia] explode all trees | 24360 |
| #8 | (Anesthetic* or Anesthesia or anaesthesia or anaesthetic or anesthetization or autoanaesthesia or autoanesthesia or narcosis or neuroanaesthesia or neuroanesthesia):ti,ab,kw | 99308 |
| #9 | #7 or #8 | 101327 |
| #10 | #6 and #9 | 205 |
| #11 | MeSH descriptor: [Aspartate Aminotransferases] explode all trees | 1139 |
| #12 | (Aspartate Aminotransferase Or Aspart* Aminotransferase* Or Aspart* Transaminase Or Aspartate Apoaminotransferase Or aspartate amino transferase Or AST Or GOT Or SGOT Or glutam* oxal* transaminase Or glutamate oxaloacetate* Or glutamate oxalacetate transaminase Or glutam* oxaloacetic* Or glutamatoxalacetate transaminase Or glutamic aspartic*) | 18992 |
| #13 | MeSH descriptor: [Alanine Transaminase] explode all trees | 1812 |
| #14 | (Alanine Transaminase Or SGPT Or Alanin* aminotransferase Or alanine amino transferase Or alanine* transaminase Or alanine transpeptidase Or glutamate pyruvate* Or glutamic pyruv* transaminase Or glutamic pyruvic aminotransferase Or glutamopyruvic transaminase Or GPT) | 12525 |
| #15 | #11 or #12 or #13 or #14 | 23307 |
| #16 | #10 and #15 | 31 |

**Web of Science**

| No. | Query | Results | Date |
| --- | --- | --- | --- |
| #1 | Hepatectomy?(Topic)?or?Hepatectomies?(Topic) | 58978 | February 12, 2025 |
| #2 | liver OR hepatic OR hepato?(Topic)?and?resection or surgery?(Topic)? | 257649 | February 12, 2025 |
| #3 | Anesthesia Or Anesthetic* Or anaesthesia Or anaesthetic Or anesthetization Or autoanaesthesia Or autoanesthesia Or narcosis Or neuroanaesthesia Or Neuroanesthesia?(Topic)? | 620030 | February 12, 2025 |
| #4 | #1 OR #2 | 276977 | February 12, 2025 |
| #5 | #3 AND #4 | 6878 | February 12, 2025 |
| #6 | Aspart*?Transaminase Or Aspartate?Apoaminotransferase Or aspartate?amino?transferase Or AST Or GOT Or SGOT Or glutam*?oxal*?transaminase? Or glutamate?oxaloacetate* Or glutamate?oxalacetate?transaminase? Or glutam*?oxaloacetic* Or glutamatoxalacetate?transaminase? Or glutamic?aspartic*?(Topic)? | 1336578 | February 12, 2025 |
| #7 | Alanine Transaminase Or SGPT Or Alanin* aminotransferase Or alanine amino transferase Or alanine* transaminase Or alanine transpeptidase Or glutamate pyruvate* Or glutamic pyruv* transaminase Or glutamic pyruvic aminotransferase Or glutamopyruvic transaminase Or GPT?(Topic) | 132539 | February 12, 2025 |
| #8 | #6 OR #7 | 1392320 | February 12, 2025 |
| #9 | #5 AND #8 | 516 | February 12, 2025 |

**Supplementary Table S1** Details of reasons for article exclusion

| **Number** | **Title** | **Reasons for article exclusion** |
| --- | --- | --- |
| 1 | Conditioning With Sevoflurane in Liver Transplantation: Results of a Multicenter Randomized Controlled Trial | Subjects do not match. |
| 2 | Dexmedetomidine exerts a protective effect on ischemia-reperfusion injury  after hepatectomy: A prospective, randomized, controlled study | Interventions do not match. |
| 3 | Diagnostic Value of Peripheral Blood miR-148a-3p in  Patients with Liver Injury After Hepatectomy Under  General Anesthesia with Propofol | Interventions do not match. |
| 4 | Effect of an anesthesia with propofol  compared with desflurane on free radical  production and liver function after partial  hepatectomy | Outcomes do not match. |
| 5 | Impact of Volatile Anesthetic Agents on Early Clinical Outcomes in  Liver Transplantation | Subjects do not match. |
| 6 | Propofol intravenous anaesthesia with desflurane  compared with desflurane alone on postoperative liver  function after living-donor liver transplantation | Subjects do not match. |
| 7 | The Effects of Desflurane and Isoflurane on Hepatic and Renal  Functions After Right Hepatectomy in Living Donors | Data cannot be extracted.  There is no baseline data, and the postoperative data is in image form, not in numerical values. |
| 8 | Thoracic Epidural Analgesia in Donor  Hepatectomy: An Analysis | Study types do not match. |
| 9 | The use of bilateral continuous erector spinae plane blocks for  postoperative analgesia after right-sided living donor  hepatectomy: A feasibility study" | Interventions do not match. |
| 10 | Effects of Isoflurane and Propofol on Hepatic and Renal Functions  and Coagulation Profile After Right Hepatectomy in Living Donors | Data are problematic.The preoperative median AST of group I is not within the listed range. |
| 11 | A Comparison of the Effect of Isoflurane and Propofol on Liver Enzymes | Study subjects do not match. |
| 12 | A prospective analysis of propofol, sevoflurane, isoflurane and desflurane on the post operative hepatic and renal functions in living donor hepatectomies | Full text is unavailable. |
| 13 | A prospective double blind study comparing the effect of desflurane and isoflurane on postoperative hepatic function after adult living donor hepatectomy | Full text is unavailable. |
| 14 | Anesthetic considerations for the patient with  liver disease | Study types do not match. |
| 15 | Comparative effect of desflurane and sevoflurane on  liver function tests of patients with impaired hepatic  function undergoing cholecystectomy: A randomized  clinical study | Study subjects do not match. |
| 16 | Dexmedetomidine reduces intestinal and hepatic injury after  hepatectomy with inflow occlusion under general  anaesthesia: a randomized controlled trial | Interventions and outcomes do not match. |
| 17 | Economic Evaluation of Pharmacologic Pre- and  Postconditioning With Sevoflurane Compared With  Total Intravenous Anesthesia in Liver Surgery:  A Cost Analysis | Outcomes do not match. |
| 18 | Effect of dexmedetomidine on hepatic  ischemia-reperfusion injury in the setting of  adult living donor liver transplantation | Interventions do not match.. |
| 19 | Effects of the Hypnotic Agent on Primary Graft Dysfunction After Liver  Transplantation | Interventions do not match. |
| 20 | Impact of Inhalational Anesthetics on Liver  Regeneration After Living Donor Hepatectomy:  A Propensity Score-Matched Analysis | References are repeated. |
| 21 | Intraoperative factors associated with delayed recovery of liver  function after hepatectomy: analysis of 1969 living donors | Interventions do not match. |
| 22 | Preconditioning Effect of Remifentanil Versus Fentanyl in Prevalence of Early  Graft Dysfunction in Patients After Liver Transplant: A Randomized Clinical  Trial | Study subjects do not match. |
| 23 | Propofol vs desflurane on the cytokine, matrix  metalloproteinase-9, and heme oxygenase-1  response during living donor liver transplantation  A pilot study | Study subjects do not match. |
| 24 | Prospective study to know the effect of different anaesthetic techniques on hepatic and renal functions in liver donors | Full text is unavailable. |
| 25 | The pharmacokinetics and safety of lidocaine in liver cancer patients  undergoing hepatic resection | Interventions do not match.. |
| 26 | The comparison of desflurane and sevoflurane  on postoperative hepatic function of infant with  biliary atresia undergoing Kasai operation | Study subjects do not match. |
| 27 | Clinical trial of sevoflurane inhalation solution in the treatment of hepaticechinococcosis in Tibetan patients at high altitude | References are repeated. |
| 28 | Comparison of hepatic ischemia/reperfusion injury caused by partial hepatectomy performed under isoflurane-fentanyl and propofol-remifentanil anesthesia | References are repeated. |
| 29 | Effect of different excipients of propofol on blood lipids and liver function during liver transplantation | Interventions do not match. |
| 30 | Influence of different anesthesia approaches on postoperative infections in liver cancer resection patients | Interventions do not match. |
| 31 | The effectiveness and safety of ultrasound-guided transversus abdominis plane block combined with laryngeal mask ventilation general anesthesia in enhanced recovery after surgery for patients with primary liver cancer | Subject, intervention, and outcome do not match. |
| 32 | Effect of improved serratus anterior plane block under ultrasound guidance on analgesia in children undergoing liver transplantation | Subject, intervention, and outcome do not match. |
| 33 | Effect of dexmedetomidine for patients undergoing precisehepatectomy in anesthesia intensive care unit | Interventions do not match. |
| 34 | Perioperative anesthetic management for recipients of orthotopic liver transplant undergoing nontransplant surgery | Study subjects do not match. |
| 35 | Analysis of initial poor graft function after orthotopic liver transplantation: Experience of an Australian Single Liver Transplantation Center | Study subjects do not match. |
| 36 | Protective effect of propofol compared with sevoflurane on liver function after hepatectomy with Pringle maneuver: a randomized clinical trial | References are repeated. |
| 37 | Application of propofol combined with sevoflurane anesthesia in staged hepatectomy liver detachment and portal vein ligation | Interventions do not match. |
| 38 | Effect of different doses of paracetamol on postoperative pain after gynecologic laparoscopy | Subject, intervention, and outcome do not match. |
| 39 | Comparison of minimal-flow sevoflurane versus desflurane anesthesia: randomized clinical trial | Outcomes do not match. |
| 40 | Anethesia care for adult live donor hepatectomy: Our experiences with 100 cases | Interventions do not match. |
| 41 | Effects of acute kidney injury after liver resection on long-term outcomes | Study types do not match. |
| 42 | The Effects of Prolonged Minimal-Flow Sevoflurane Anesthesia on Postoperative Hepatic and Renal Function | Study subjects do not match. |
| 43 | A Comparison of Liver Function After Hepatectomy with Inﬂow Occlusion Between Sevoﬂurane and Propofol Anesthesia | Data cannot be extracted.Postoperative data is in image form, without specific numerical values. |
| 44 | Effects of Desflurane and Isoflurane on Hepatic and Renal Functions  and Coagulation Profile During Donor Hepatectomy | Data cannot be extracted.Postoperative data is in image form, without specific numerical values. |
| 45 | The effects of desflurane and sevoflurane on hepatic and  renal functions after right hepatectomy in living donors | Data cannot be extracted.Postoperative data is in image form, without specific numerical values. |
| 46 | Impact of Inhalational Anesthetics on Liver  Regeneration After Living Donor Hepatectomy:  A Propensity Score-Matched Analysis | Outcomes do not match. |
| 47 | Propofol/dexmedetomidine Versus Desflaurane  Effects on Post Hepatectomy Hepatocellular Injury | Intervention measures cannot form a loop, nor can they be analyzed in such a manner. |
| 48 | Comparison of effects of different anesthesia  methods on immune function and liver function of  liver cancer patients after operation | Data cannot be extracted.The data format is inconsistent with other studies, making unified comparison impossible. |
| 49 | Protection of Pharmacological Postconditioning in Liver Surgery Results of a Prospective Randomized Controlled Trial | Interventions do not match. |
| 50 | Dexmedetomidine ameliorates liver injury and maintains liver function in patients with  hepatocellular carcinoma after hepatectomy: a retrospective cohort study with propensity  score matching | Data cannot be extracted.Postoperative data is in image form, without specific numerical values. |

**Supplementary Table S2** Baseline characteristics

| **Research characteristics** | | | | | **Patient characteristics** | | | | |
| --- | --- | --- | --- | --- | --- | --- | --- | --- | --- |
| **Study** | **Country** | **Time** | **Type of Study** | **intervetion** | **Sample size (M/F)** | **Age** | **Disease** | **Anesthesia method** | **Ending Measurement Time** |
|  |  |  |  |  |  |  |  |  |  |
| Yang et al.2010 | China | 2008.12-2009.5 | Double -blind RCT | Isoflurane(1-2%,2L/min) | 30(22/8) | 52±9.1 | Cirrhosis | Inhalation anesthesia for epidural block | 1 day after surgery |
|  |  |  |  | Propofol(1.5-2ug/kg) | 30(21/9) | 53.6±9.5 |  | Intravenous anesthesia for epidural block |  |
| Xue et al.2006 | China |  | RCT | Propofol(200mg)+ Remifentanil(0.2mg) | 15 (9/6) | 53.71±13.71 | Liver cancer | Combined epidural and general anesthesia | 1 day after surgery |
|  |  |  |  | Isoflurane+ Fentanyl | 15 (8/7) | 49.89±16.93 |  | Simple static-inhalation combined general anesthesia |  |
| Wang et al.2019 | China | 2016.3-2018-7 | Retrospective analysis | Propofol(2.0-2.5 mg/kg) | 30(22/8) | 45.94±10.67 | Cirrhosis | Intravenous anesthesia | after the portal triad clamping |
|  |  |  |  | Propofol(2.0-2.5 mg/kg)+Remifentanil(0.5-1ug/kg) | 38 (31/7) | 47.25±9.69 |  | Intravenous anesthesia |  |
| Truong Minh et al.2019 | France | 2013.3-2014.8 | Retrospective analysis | Sevoflurane(1.2%-2.5%) | 67 (34/33) | 60.71±3.4 | Liver resection with intermittent triple portal vein occlusion | Inhalation anesthesia | 1 day after surgery |
|  |  |  |  | Propofol(2-3mg/kg)+Sevoflurane(1.5MAC) | 27 (17/10) | 64.37±2.75 |  | Intravenous anesthesia combined with inhalation anesthesia |  |
|  |  |  |  | Propofol(2-3mg/kg) | 26 (15/11) | 62.36±3.79 |  | Intravenous anesthesia |  |
| Slankamenac et al.2012 | Swiss | 2005.1.1-2007.12.31 | Retrospective analysis | Sevoflurane(1-2.5%) | 141 (88/53) | 59.2±14.8 | Liver resection with inflow obstruction | Inhalation anesthesia | peak |
|  |  |  |  | Propofol((plasma target concentration of 2-4ug/mL) | 86 (49/37) | 56.3±12.7 |  | Intravenous anesthesia |  |
| Shen et al.2015 | China |  | RCT | Sevoflurane (1MAC) | 30 |  | Liver cirrhosis combined with liver cancer | Intravenous anesthesia combined with inhalation anesthesia | 1 day after surgery |
|  |  |  |  | Isoflurane (1MAC) | 30 |  |  | Intravenous anesthesia combined with inhalation anesthesia |  |
| Rabie et al.2006 | KingdomSaudi Arabia | 2002.2-2004.8 | RCT | Propofol(2-3mg/kg)+Isoflurane(0.8-1.2%;FiO2=0.4-0.5)+Fentanyl(2ug/kg) | 10(9/1) | 26.8±5.3 | Liver Transplantation | Intravenous anesthesia combined with inhalation anesthesia | 1 day after surgery |
|  |  |  |  | Propofol(2-3mg/kg)+Sufentanil(0.2mg/kg) | 10 (8/2) | 24.6±4.5 |  | Intravenous anesthesia |  |
| Petrov et al.2012 | Bulgaria | 2009.1-2010.6 | Retrospective analysis | Sevoflurane(2.0%) | 17 (6/11) | 62±5.3 | Liver resection | Inhalation anesthesia | 1 day after surgery |
|  |  |  |  | Isoflurane(1.5%) | 25 (11/14) | 62±5.7 |  | Inhalation anesthesia |  |
| Nishiyama et al.2004 | Japan |  | Double -blind RCT | Sevoflurane(4-6ml) | 20 (20/0) | 59±12 | Cirrhosis | Epidural block combined with inhalation anesthesia | 1 day after surgery |
|  |  |  |  | Isoflurane(4-6ml) | 20 (20/0) | 55±14 |  | Epidural block combined with inhalation anesthesia |  |
| Lyu et al.2019 | China | 2017.9-2018.7 | RCT | Sevoflurane(2%) | 25 (13/12) | 43.7±7.4 | Alveolar liver hydatid | Inhalation anesthesia | 1 day after surgery |
|  |  |  |  | Propofol(1.5-2.5ug/ml)+Remifentanil(0.2ug/kg/min) | 25 (11/14) | 45.1±8.2 |  | Intravenous anesthesia |  |
| Liao et al.2020 | China | 2016.2-2018.6 | Retrospective analysis | Propofol(4-8mg/kg/h)+Sufentanil(0.2-0.3 ug/kg/h) | 72 (39/33) | 48.3±6.3 | Liver cancer | Intravenous anesthesia | postoperative |
|  |  |  |  | Propofol(4-8mg/kg/h)+Remifentanil(2-4ng/kg/h) | 65 (38/27) | 46.9±5.6 |  | Intravenous anesthesia |  |
| Jiang et al.2017 | China | 2014.1-2016.1 | RCT | Propofol(6.5-9.0mg/kg/h)+Isoflurane(2-3%) | 30(17/13) | 54.87±3.76 | Liver cancer | Intravenous anesthesia combined with inhalation anesthesia | 1 day after surgery |
|  |  |  |  |  |  |  |  |  |  |
|  |  |  |  | Propofol(7-10 mg/kg/h)+Remifentanil(0.3-0.5 g/kg/min) | 30(15/15) | 53.43±4.12 |  | Intravenous anesthesia |  |
|  |  |  |  |  |  |  |  |  |  |
| Liu et al.2008 | China |  | RCT | Isoflurane(1-2%) | 30(16/14) | 46±9 | Liver resection | Inhalation anesthesia | 10min after hepatic portal opening |
|  |  |  |  |  |  |  |  |  |  |
|  |  |  |  | Propofol(4-6mg/kg/h) | 30(14/16) | 44±13 |  | Intravenous anesthesia |  |
|  |  |  |  |  |  |  |  |  |  |
| Beck-Schimmer et al.2008 | Swiss | 2006.4-2007.11 | Double -blind RCT | Propofol(2-4g/ml)+Sevoflurane(3.2%) | 30(16/14) | 54.23±12.74 | Liver resection with inflow obstruction | Intravenous anesthesia combined with inhalation anesthesia | peak |
|  |  |  |  |  |  |  |  |  |  |
|  |  |  |  | Propofol(2-4g/ml) | 34(19/15) | 57.82±12.82 |  | Intravenous anesthesia |  |
|  |  |  |  |  |  |  |  |  |  |
| Gao et al.2009 | China | 2003.10-2006.10 | RCT | Propofol(3.5mg/L)+Remifentanil(4.2ug/L) | 15(8/7) | 51±9 | Liver cancer, hepatic hemangioma | Intravenous anesthesia | 1 day after surgery |
|  |  |  |  |  |  |  |  |  |  |
|  |  |  |  | Isoflurane(1.5-2.5%)+Fentanyl(2ug/kg) | 15(9/6) | 53±8 |  | Intravenous anesthesia combined with inhalation anesthesia |  |
|  |  |  |  |  |  |  |  |  |  |
| Beck-Schimmer et al.2012 | Swiss | 2008.1-2010.9 | RCT | Propofol(2-6ug/mL)+Sevoflurane(3.2%)+Pringle | 48(26/22) | 60.9101±3.8158 | Liver tumors | Intravenous anesthesia combined with inhalation anesthesia | peak |
|  |  |  |  |  |  |  |  |  |  |
|  |  |  |  | Propofol(2-6ug/mL) | 67 (45/22) | 60.13±3.39 |  | Intravenous anesthesia |  |
| Matsumi et al.2023 | Japan | 2019.1.11-2020.11.19 | RCT | Sevoflurane(0.6-2%) | 28(19/9) | 66.3±12.3 | Liver metastasis resection | Inhalation anesthesia | peak |
|  |  |  |  | Propofol(BIS:30-70) | 28(20/8) | 64.7±10.1 |  | Intravenous anesthesia |  |
| Xu et al.2021 | China | 2007.11-2017.12 | Retrospective analysis | Propofol(plasma concentration, 3 µg/ml) | 21(11/10) | 44.91±5.32 | Liver cancer | Intravenous anesthesia | postoperative |
|  |  |  |  |  |  |  |  |  |  |
|  |  |  |  | Propofol(plasma concentration, 3 µg/ml)+Sevoflurane(0.5MAC) | 19(10/9) | 46.33±5.64 |  | Intravenous anesthesia combined with inhalation anesthesia |  |

| Jiang et al.2018 | China | 2016.01-2017.12 | RCT | Dexmedetomidine(0.5ug/kg/h)+Propofol(0.5mg/kg/h) | 20(14/6) | 46.7±10.6 | Precision liver resection | ntravenous anesthesia combined with inhalation anesthesia | postoperative |
| --- | --- | --- | --- | --- | --- | --- | --- | --- | --- |
|  |  |  |  | Propofol(0.5mg/kg/h) | 20(16/4) | 44.6±9.2 |  |  |  |

**Supplementary Table S3** Scura ranking table

| **Measure** | **ALT** | **AST** |
| --- | --- | --- |
| Isoflurane | 0.38 | 0.38 |
| Isoflurane_Fentanyl | 0.10 | 0.10 |
| Propofol | 0.52 | 0.52 |
| Propofol_Isoflurane | 0.38 | 0.38 |
| Propofol_Isoflurane_Fentanyl | 0.62 | 0.62 |
| Propofol_Remifentanil | 0.53 | 0.53 |
| Propofol_Sevoflurane | 0.72 | 0.72 |
| Propofol_Sufentanil | 0.57 | 0.56 |
| Sevoflurane | 0.59 | 0.58 |
| Propofol_Dexmedetomidine | 0.59 | 0.59 |

**Supplementary Table S4** Grade assessments

| Comparison | Study design | limitations | Indirectness | Inconsistency | Imprecision | Risk of bias | Certainty |
| --- | --- | --- | --- | --- | --- | --- | --- |
| Isoflurane  Vs  Isoflurane_Fentanyl | RCT | Not serious | Not serious | Not serious | Not serious | Serious | ⊕⊕⊕◯ |
| Isoflurane  Vs  Propofol | RCT | Not serious | Not serious | Not serious | Not serious | Not serious | ⊕⊕⊕⊕ |
| Isoflurane  Vs Propofol_Isoflurane | RCT | Not serious | Not serious | Not serious | Not serious | Serious | ⊕⊕⊕◯ |
| Isoflurane  Vs Propofol_Isoflurane_Fentanyl | RCT | Not serious | Not serious | Not serious | Not serious | Serious | ⊕⊕⊕◯ |
| Isoflurane  Vs Propofol_Remifentanil | RCT | Not serious | Not serious | Not serious | Not serious | Serious | ⊕⊕⊕◯ |
| Isoflurane  Vs Propofol_Sevoflurane | RCT | Not serious | Not serious | Not serious | Not serious | Serious | ⊕⊕⊕◯ |
| Isoflurane  Vs Propofol_Sufentanil | RCT | Not serious | Not serious | Not serious | Not serious | Serious | ⊕⊕⊕◯ |
| Isoflurane  Vs Sevoflurane | RCT | Not serious | Not serious | Not serious | Not serious | Not serious | ⊕⊕⊕⊕ |
| Isoflurane_Fentanyl Vs Propofol | RCT | Not serious | Not serious | Not serious | Not serious | Serious | ⊕⊕⊕◯ |
| Isoflurane_Fentanyl Vs Propofol_Isoflurane | RCT | Not serious | Not serious | Not serious | Not serious | Serious | ⊕⊕⊕◯ |
| Isoflurane_Fentanyl Vs Propofol_Isoflurane_Fentanyl | RCT | Not serious | Not serious | Not serious | Not serious | Serious | ⊕⊕⊕◯ |
| Isoflurane_Fentanyl Vs Propofol_Remifentanil | RCT | Not serious | Not serious | Not serious | Not serious | Serious | ⊕⊕⊕◯ |
| Isoflurane_Fentanyl Vs Propofol_Sevoflurane | RCT | Not serious | Not serious | Not serious | Not serious | Serious | ⊕⊕⊕◯ |
| Isoflurane_Fentanyl Vs Propofol_Sufentanil | RCT | Not serious | Not serious | Not serious | Not serious | Serious | ⊕⊕⊕◯ |
| Isoflurane_Fentanyl Vs Sevoflurane | RCT | Not serious | Not serious | Not serious | Not serious | Serious | ⊕⊕⊕◯ |
| Propofol Vs Propofol_Isoflurane | RCT | Not serious | Not serious | Not serious | Not serious | Serious | ⊕⊕⊕◯ |
| Propofol Vs Propofol_Isoflurane_Fentanyl | RCT | Not serious | Not serious | Not serious | Not serious | Serious | ⊕⊕⊕◯ |
| Propofol Vs Propofol_Remifentanil | Retrospective analysis | Serious | Not serious | Not serious | Not serious | Serious | ⊕⊕◯◯ |
| Propofol Vs Propofol_Sevoflurane | RCT | Not serious | Not serious | Not serious | Not serious | Serious | ⊕⊕⊕◯ |
| Propofol Vs Propofol_Sufentanil | RCT | Not serious | Not serious | Not serious | Not serious | Serious | ⊕⊕⊕◯ |
| Propofol Vs Sevoflurane | Retrospective analysis | Serious | Not serious | Not serious | Not serious | Not serious | ⊕⊕⊕◯ |
| Propofol_Isoflurane Vs Propofol_Isoflurane_Fentanyl | RCT | Not serious | Not serious | Not serious | Not serious | Serious | ⊕⊕⊕◯ |
| Propofol_Isoflurane Vs Propofol_Remifentanil | RCT | Not serious | Not serious | Not serious | Not serious | Serious | ⊕⊕⊕◯ |
| Propofol_Isoflurane Vs Propofol_Sevoflurane | RCT | Not serious | Not serious | Not serious | Not serious | Serious | ⊕⊕⊕◯ |
| Propofol_Isoflurane Vs Propofol_Sufentanil | RCT | Not serious | Not serious | Not serious | Not serious | Serious | ⊕⊕⊕◯ |
| Propofol_Isoflurane Vs Sevoflurane | RCT | Not serious | Not serious | Not serious | Not serious | Serious | ⊕⊕⊕◯ |
| Propofol_Isoflurane_Fentanyl Vs Propofol_Remifentanil | RCT | Not serious | Not serious | Not serious | Not serious | Serious | ⊕⊕⊕◯ |
| Propofol_Isoflurane_Fentanyl Vs Propofol_Sevoflurane | RCT | Not serious | Not serious | Not serious | Not serious | Serious | ⊕⊕⊕◯ |
| Propofol_Isoflurane_Fentanyl Vs Propofol_Sufentanil | RCT | Not serious | Not serious | Not serious | Not serious | Serious | ⊕⊕⊕◯ |
| Propofol_Isoflurane_Fentanyl Vs Sevoflurane | RCT | Not serious | Not serious | Not serious | Not serious | Serious | ⊕⊕⊕◯ |
| Propofol_Remifentanil Vs Propofol_Sevoflurane | RCT | Not serious | Not serious | Not serious | Not serious | Serious | ⊕⊕⊕◯ |
| Propofol_Remifentanil Vs Propofol_Sufentanil | Retrospective analysis | Serious | Not serious | Not serious | Not serious | Serious | ⊕⊕◯◯ |
| Propofol_Remifentanil Vs Sevoflurane | RCT | Not serious | Not serious | Not serious | Not serious | Serious | ⊕⊕⊕◯ |
| Propofol_Sevoflurane Vs Propofol_Sufentanil | RCT | Not serious | Not serious | Not serious | Not serious | Serious | ⊕⊕⊕◯ |
| Propofol_Sevoflurane Vs Sevoflurane | Retrospective analysis | Serious | Not serious | Not serious | Not serious | Serious | ⊕⊕◯◯ |
| Propofol_Sufentanil Vs Sevoflurane | RCT | Not serious | Not serious | Not serious | Not serious | Serious | ⊕⊕⊕◯ |
| Propofol_Dexmedetomidine Vs Propofol | RCT | Not serious | Not serious | Not serious | Not serious | Serious | ⊕⊕⊕◯ |

**Supplementary Table S5** Scura ranking table of non-cirrhotic

| **Measure** | **ALT** | **AST** |
| --- | --- | --- |
| Isoflurane | 0.40 | 0.44 |
| Isoflurane_Fentanyl | 0.13 | 0.10 |
| Propofol | 0.65 | 0.70 |
| Propofol_Isoflurane | 0.40 | 0.41 |
| Propofol_Isoflurane_Fentanyl | 0.57 | 0.43 |
| Propofol_Remifentanil | 0.50 | 0.50 |
| Propofol_Sevoflurane | 0.76 | 0.84 |
| Propofol_Sufentanil | 0.52 | 0.51 |
| Sevoflurane | 0.56 | 0.56 |

**Supplementary Table S6a** League Table for Sensitivity Analysis 1 (AST)

| **Isoflurane** | 119.4 (-196.54, 502.9) | -63.26 (-243.35, 98.94) | 17.64 (-360.51, 403.78) | -41.07 (-505.84, 428.54) | -14.49 (-303.52, 282.21) | -93.1 (-314.37, 95.34) | -22.81 (-407.74,366.77) | -39.5 (-187.23, 115.6) |
| --- | --- | --- | --- | --- | --- | --- | --- | --- |
|  | **Isoflurane_Fentanyl** | -184.41 (-569.18, 120.4) | -102.23 (-449.38, 183.67) | -160.54 (-603.52, 222.79) | -135.95 (-357.41, 32.43) | -215.12 (-623.13, 99.63) | -142.34 (-491.85, 143.41) | -158.46 (-505.17, 127.78) |
|  |  | **Propofol** | 80.17 (-285.69, 470.06) | 22.56 (-429.01, 494.77) | 48.83 (-224.02, 346.3) | -29.99 (-167.89, 93.31) | 39.55 (-329.65, 431.44) | 24.7 (-101.56, 172.02) |
|  |  |  | **Propofol_Isoflurane** | -58.8 (-499.21, 381.61) | -31.45 (-280.49, 217.94) | -110.11 (-521.57, 261.33) | -40.04 (-395.36, 313.53) | -56.69 (-407.59, 295.36) |
|  |  |  |  | **Propofol_Isoflurane_Fentanyl** | 26.84 (-336.98, 389.18) | -51.87 (-543.36, 403.87) | 17.96 (-245.8, 278.71) | 2.07 (-438.01, 446.11) |
|  |  |  |  |  | **Propofol_Remifentanil** | -78.66 (-400.37, 205.16) | -8.55 (-258.94, 242.73) | -24.88 (-274.73, 225.14) |
|  |  |  |  |  |  | **Propofol_Sevoflurane** | 22.21 (-200.55, 270.89) | 23.35 (-76.3, 159.86) |
|  |  |  |  |  |  |  | **Propofol_Sufentanil** | 0.08 (-211.6, 225.18) |
|  |  |  |  |  |  |  |  | **Sevoflurane** |

**Supplementary Table S6b** League Table for Sensitivity Analysis 1 (ALT)

| **Isoflurane** |  |  |  |  |  |  |  |  |
| --- | --- | --- | --- | --- | --- | --- | --- | --- |
| -163.65 (-554.16, 167.5) | **Isoflurane_Fentanyl** |  |  |  |  |  |  |  |
| 75.51 (-97.24, 253.81) | 239.69 (-80.73, 625.45) | **Propofol** |  |  |  |  |  |  |
| -28.85 (-427.1, 369.12) | 135.02 (-161.02, 489.81) | -103.76 (-497.68, 280.82) | **Propofol_Isoflurane** |  |  |  |  |  |
| -24.96 (-501.33, 450.6) | 138.63 (-255.28, 586.25) | -100.21 (-575.64, 367.39) | 3.55 (-447.06, 453.03) | **Propofol_Isoflurane_Fentanyl** |  |  |  |  |
| 4.16 (-297.84, 305.52) | 169.78 (-5.46, 394.77) | -71.29 (-367, 218.56) | 32.82 (-225.11, 289.74) | 28.79 (-340.65, 401.24) | **Propofol_Remifentanil** |  |  |  |
| 121.24 (-77.11, 343.55) | 285.59 (-42.26, 696.08) | 45.7 (-77.64, 187.55) | 149.51 (-242.03, 565.21) | 146.48 (-325.82, 642.92) | 116.75 (-181.32, 439.46) | **Propofol_Sevoflurane** |  |  |
| 7.79 (-388.11, 401.42) | 171.8 (-126.56, 526.13) | -67.96 (-460.29, 319.08) | 35.9 (-328.67, 401.18) | 32.74 (-234.09, 298.61) | 3.43 (-254.93, 261.97) | -113.87 (-529.98, 279.26) | **Propofol_Sufentanil** |  |
| 35.16 (-122.18, 190.01) | 198.75 (-98.62, 553.5) | -40.51 (-182.15, 93.36) | 64.1 (-301.91, 427.13) | 60.22 (-391, 512) | 31.05 (-227.59, 287.96) | -86.4 (-272.3, 74.28) | 27.89 (-338.4, 389.71) | **Sevoflurane** |

**Supplementary Table S7** Scura ranking table of enzyme measurement time

| **Measure** | **ALT** | **AST** |
| --- | --- | --- |
| Isoflurane | 0.44 | 0.44 |
| Isoflurane_Fentanyl | 0.10 | 0.10 |
| Propofol | 0.70 | 0.70 |
| Propofol_Isoflurane | 0.41 | 0.41 |
| Propofol_Isoflurane_Fentanyl | 0.43 | 0.43 |
| Propofol_Remifentanil | 0.50 | 0.50 |
| Propofol_Sevoflurane | 0.84 | 0.84 |
| Propofol_Sufentanil | 0.52 | 0.52 |
| Sevoflurane | 0.56 | 0.56 |

**Supplementary Table S8a** League Table for Sensitivity Analysis 2 (AST)

| **Isoflurane** | 163.23 (-168.68, 553.83) | -75.68 (-253.34, 95.7) | 28.42 (-366.96, 425.71) | 25.08 (-450.57, 503.18) | -4.09 (-305.23, 297.52) | -121.27 (-342.46, 75.15) | -7.89 (-402.76, 388.24) | -34.92 (-190.3, 121.31) |
| --- | --- | --- | --- | --- | --- | --- | --- | --- |
|  | **Isoflurane_Fentanyl** | -239.87 (-623.26, 82.42) | -134.94 (-488.96, 161.47) | -138.04 (-586.23, 250.85) | -169.33 (-394.41, 5.2) | -286.08 (-695.02, 43.7) | -171.4 (-524.53, 125.77) | -198.72 (-550.91, 98.32) |
|  |  | **Propofol** | 104.14 (-283.8, 496.18) | 100.72 (-366.55, 575.57) | 71.45 (-216.59, 365.98) | -45.7 (-185.5, 78.28) | 67.74 (-319.1, 458.93) | 40.91 (-93, 181.02) |
|  |  |  | **Propofol_Isoflurane** | -3.37 (-451.78, 446.72) | -32.78 (-290.74, 226) | -149.38 (-566.09, 243.57) | -36.61 (-400.38, 328.64) | -63.21 (-424.93, 302.9) |
|  |  |  |  | **Propofol_Isoflurane_Fentanyl** | -28.98 (-398.77, 339.95) | -146.05 (-641.5, 324.1) | -32.66 (-297.26, 232.37) | -59.83 (-510.36, 391.23) |
|  |  |  |  |  | **Propofol_Remifentanil** | -117.01 (-439.09, 180.46) | -3.8 (-260.25, 254.24) | -31.11 (-285.92, 227.16) |
|  |  |  |  |  |  | **Propofol_Sevoflurane** | 113.08 (-277.25, 527.22) | 86.65 (-73.42, 272.22) |
|  |  |  |  |  |  |  | **Propofol_Sufentanil** | -26.88 (-388.99, 335.71) |
|  |  |  |  |  |  |  |  | **Sevoflurane** |

**Supplementary Table S8b** League Table for Sensitivity Analysis 2 (ALT)

| **Isoflurane** |  |  |  |  |  |  |  |  |
| --- | --- | --- | --- | --- | --- | --- | --- | --- |
| -164.34 (-562.88, 172.52) | **Isoflurane_Fentanyl** |  |  |  |  |  |  |  |
| 76.16 (-98.54, 254.31) | 241.12 (-86.54, 629.68) | **Propofol** |  |  |  |  |  |  |
| -29.39 (-430.06, 370.99) | 135.76 (-164.74, 492.78) | -105.14 (-503.34, 285.16) | **Propofol_Isoflurane** |  |  |  |  |  |
| -24.93 (-505.54, 454.8) | 139.28 (-256.25, 592.54) | -100.73 (-579.12, 372.17) | 3.73 (-451.27, 460.28) | **Propofol_Isoflurane_Fentanyl** |  |  |  |  |
| 3.56 (-303.12, 308.08) | 170.32 (-6.42, 397.8) | -71.98 (-371.7, 221.49) | 33.05 (-226.66, 292.12) | 28.8 (-344.52, 402.05) | **Propofol_Remifentanil** |  |  |  |
| 121.43 (-78.59, 345.25) | 287.26 (-47.59, 702.44) | 45.89 (-79.34, 188.66) | 151.05 (-246.6, 569.07) | 146.97 (-327.85, 645.3) | 117.51 (-184.27, 443.73) | **Propofol_Sevoflurane** |  |  |
| 7.74 (-393.39, 407.56) | 172.19 (-126.54, 530.62) | -68.21 (-463.77, 321.87) | 36.63 (-330.65, 404.48) | 32.99 (-235.48, 299.66) | 3.67 (-254.95, 263.47) | -114.19 (-532.91, 283.25) | **Propofol_Sufentanil** |  |
| 34.92 (-123.18, 190.63) | 199.54 (-102.56, 558.36) | -41.43 (-184.18, 96) | 64.22 (-305.31, 428.56) | 59.74 (-394.9, 515.4) | 31.18 (-230.93, 291.84) | -87.22 (-275.21, 77.6) | 27.33 (-340.43, 395.9) | **Sevoflurane** |

**Supplementary Table S9** Scura ranking table of RCT

| **Measure** | **ALT** | **AST** |
| --- | --- | --- |
| Isoflurane | 0.53 | 0.51 |
| Isoflurane_Fentanyl | 0.15 | 0.15 |
| Propofol | 0.56 | 0.63 |
| Propofol_Isoflurane | 0.42 | 0.39 |
| Propofol_Remifentanil | 0.48 | 0.45 |
| Propofol_Sevoflurane | 0.77 | 0.77 |
| Sevoflurane | 0.50 | 0.46 |
| Propofol_Dexmedetomidine | 0.59 | 0.63 |

**Supplementary Table S10a** League Table for Sensitivity Analysis 3 (AST)

| **Isoflurane** | 206.47 (-279.3, 747.41) | -43.31 (-279.16, 182.13) | -56.49 (-499.79, 375.91) | 78.94 (-484.39, 662.15) | 47.39 (-379.59, 491.81) | -116.52 (-501.68, 231.65) | 23.25 (-199.74, 261.03) |
| --- | --- | --- | --- | --- | --- | --- | --- |
|  | **Isoflurane_Fentanyl** | -249.66 (-814.38, 250.97) | -262.58 (-945.31, 354.14) | -126.36 (-608.02, 315.45) | -159.01 (-452.43, 96.4) | -323.36 (-976.77, 237) | -182.73 (-661.86, 256.92) |
|  |  | **Propofol** | -13.21 (-383.19, 357.67) | 122.63 (-455.62, 727.01) | 90.74 (-353.73, 561.94) | -73.99 (-371.73, 199.09) | 66.88 (-190.27, 348.57) |
|  |  |  | **Propofol_Dexmedetomidine** | 135.64 (-549.59, 847.19) | 103.64 (-470.64, 706.83) | -59.42 (-543.48, 393.26) | 80.28 (-368.58, 550.21) |
|  |  |  |  | **Propofol_Isoflurane** | -32.04 (-402.33, 336.65) | -195.24 (-883.88, 431.47) | -56.32 (-584.49, 466.11) |
|  |  |  |  |  | **Propofol_Remifentanil** | -163.91 (-734.49, 347.74) | -24.3 (-394.09, 345.59) |
|  |  |  |  |  |  | **Propofol_Sevoflurane** | 140.85 (-229.17, 560.58) |
|  |  |  |  |  |  |  | **Sevoflurane** |

**Supplementary Table S10b** League Table for Sensitivity Analysis 3 (ALT)

| **Isoflurane** |  |  |  |  |  |  |  |
| --- | --- | --- | --- | --- | --- | --- | --- |
| -242.04 (-854.4,319.69) | **Isoflurane_Fentanyl** |  |  |  |  |  |  |
| 16.25 (-259.68, 274.88) | 257.89 (-334.37, 882.6) | **Propofol** |  |  |  |  |  |
| 35.16 (-474.14, 527.31) | 276.54 (-448.51,1037.78) | 18.73 (-404.11, 442.29) | **Propofol_Dexmedetomidine** |  |  |  |  |
| -78.88 (-741.97,566.28) | 162.35 (-343.89, 704.53) | -95.18 (-770.17,576.46) | -114.28 (-913.59, 678.48) | **Propofol_Isoflurane** |  |  |  |
| -45.73 (-551.52,444.49) | 196.36 (-96.4, 524.41) | -62.11 (-584.81, 461.71) | -81.58 (-755.13, 598.11) | 33.55 (-386.85, 456.22) | **Propofol_Remifentanil** |  |  |
| 126.27 (-283.23,538.91) | 368.72 (-288.19,1080.41) | 110.61 (-194.99, 432.07) | 91.2 (-425.15, 628.94) | 205.62 (-524.89, 959.18) | 172.64 (-427.87, 790.89) | **Propofol_Sevoflurane** |  |
| -15.77 (-283.49,239.92) | 226.55 (-283.07, 771.26) | -31.25 (-335.63, 274.94) | -50.3 (-569.67, 469.7) | 63.31 (-535.36, 663.41) | 30.07 (-393.86, 458.03) | -142.32 (-586.8, 285.76) | **Sevoflurane** |


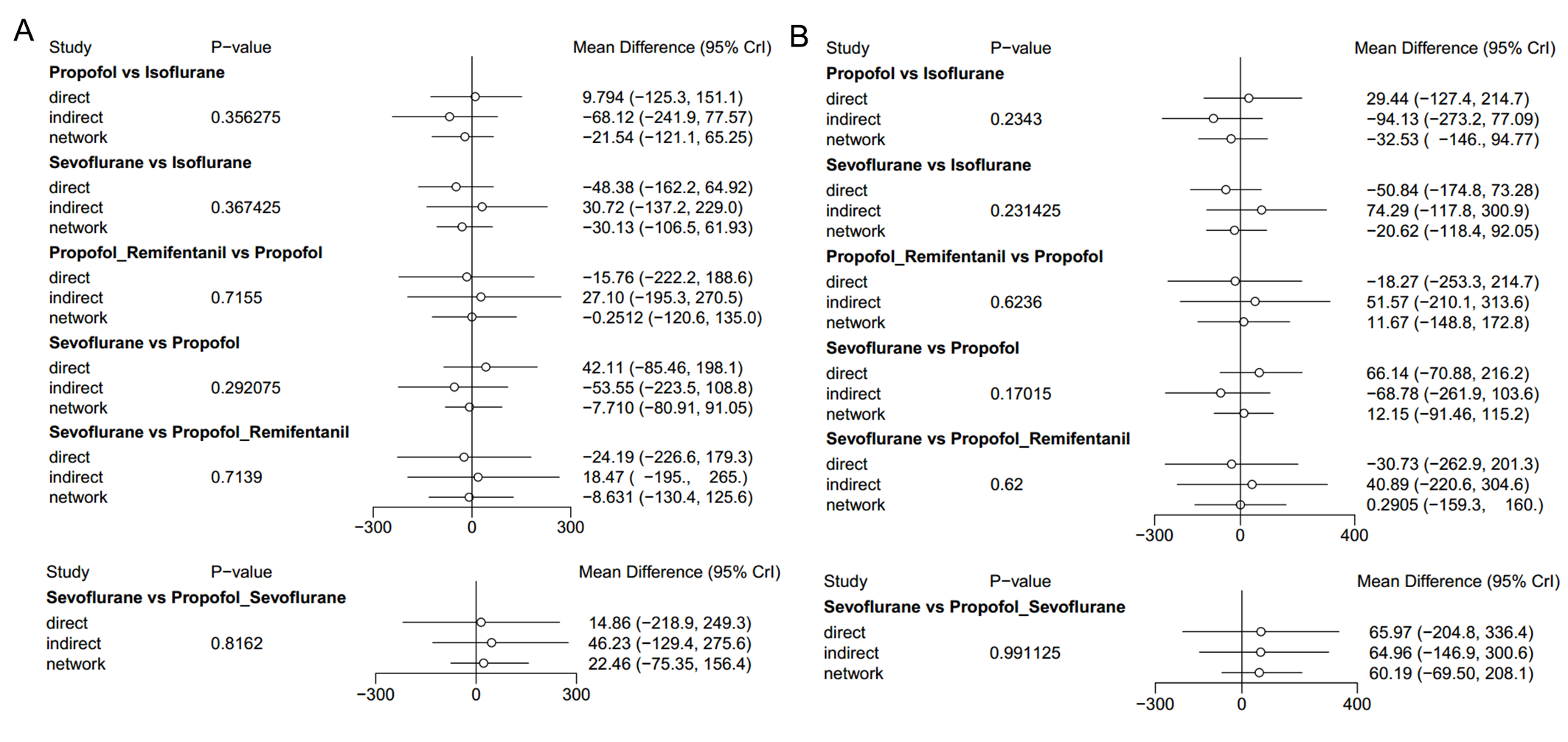


**Supplementary Figure S1** Results of (A) AST and (B) ALT node splitting analysis


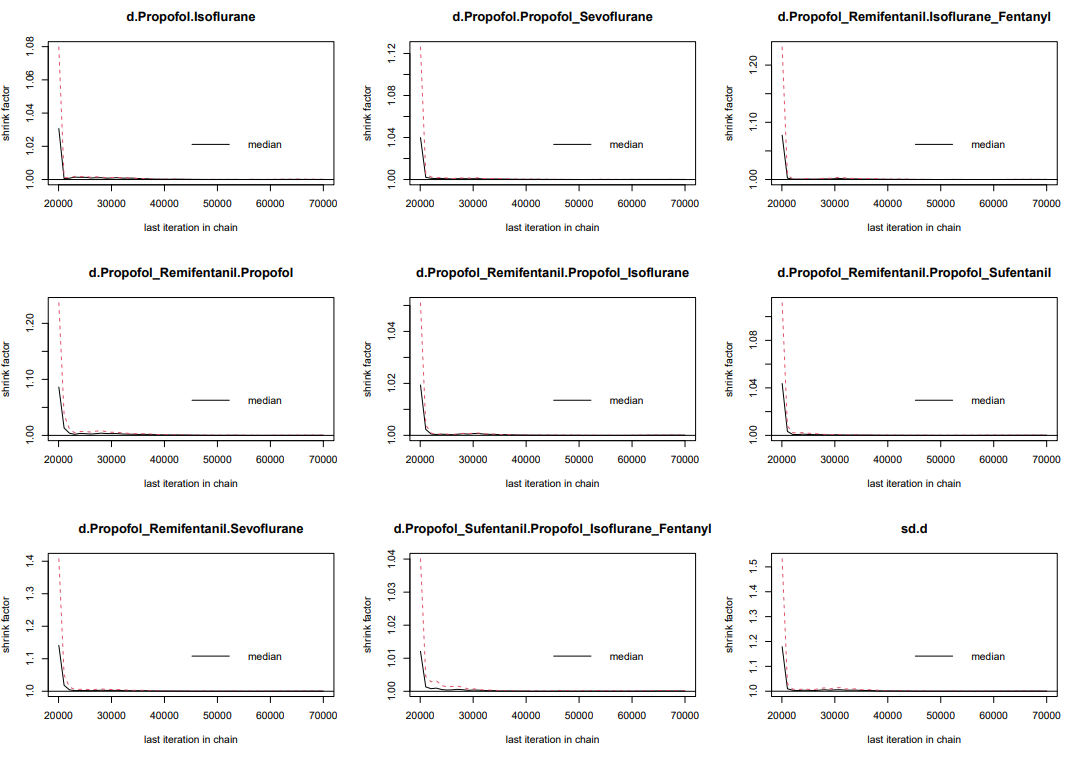


**Supplementary Figure S2** Results of Convergence diagnostics for MCMC (AST)


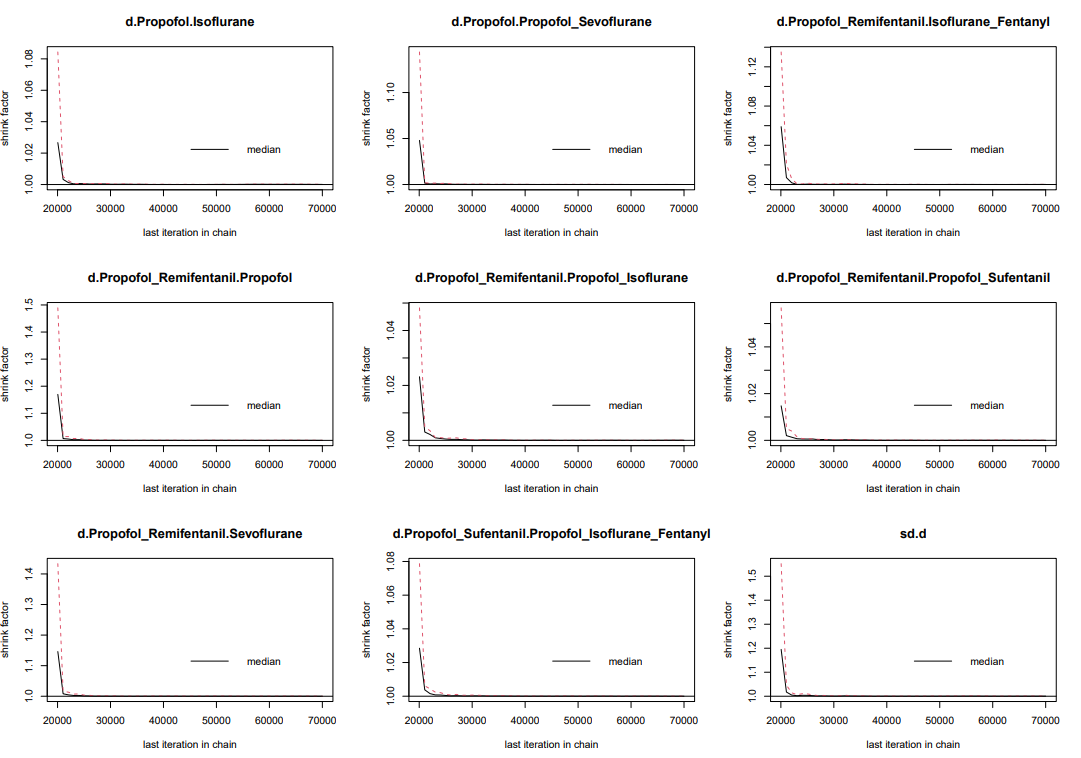


**Supplementary Figure S3** Results of Convergence diagnostics for MCMC (ALT)


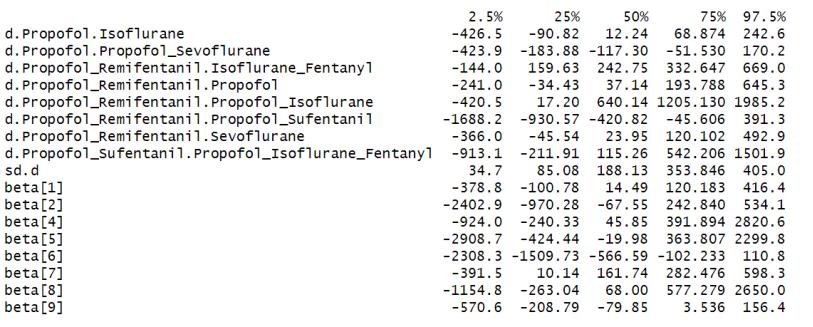


**Supplementary Figure S4** Meta-regression analysis for type of study


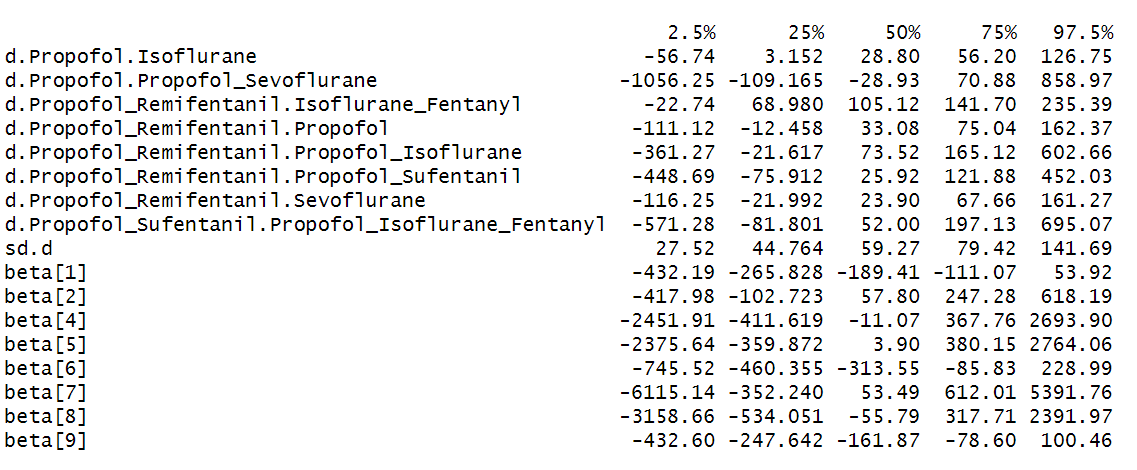


**Supplementary Figure S5** Meta-regression analysis for anesthesia method
